# An Efficient Damage-Plasticity DEM Contact Model for Highly Porous Rocks

**DOI:** 10.1007/s00603-025-04411-0

**Published:** 2025-02-19

**Authors:** Jinhui Zheng, Matteo Oryem Ciantia

**Affiliations:** 1 School of Science and Engineering, University of Dundee, Dundee, UK; 2 Department of Earth and Environmental Sciences, University of Milano Bicocca, Milan, Italy

**Keywords:** Discrete-element modelling, Soft rocks, Chalk, Soil/structure interaction

## Abstract

A novel discrete element method (DEM) model is proposed to better reproduce the behaviour of porous soft rocks. With the final goal of simulating pile penetration problems efficiency and scalability are two underlining features. The contact model is based on the macro-element theory and employs damage laws to govern the plastic deformations developing at the microscale. To attain (i) high porosity states, (ii) represent irregular shaped grains and (iii) incorporate the physical presence of bond fragments, the model is cast within a far-field interaction framework allowing for non-overlapping particles to transmit forces. After presenting a calibration procedure, the model is used to replicate the behaviour of Maastricht calcarenite. In particular, the mechanical response of this calcarenite is explored within the critical state theory framework. Finally, the efficiency, performance and scalability of the model is tested by simulating physical model experiments of cone-ended penetration tests in Maastricht calcarenite from the literature. To boost efficiency of the 3D numerical simulations, a coupled DEM-FDM (Finite Differential Method) framework is used. The good fit between the experimental and numerical results suggest that the new model can be used to unveil microscopic mechanism controlling the macroscopic response of soft-rock/structure interaction problems.

## Highlights

- A new plasticity bond damage model for highly porous, soft rocks is proposed.
- A far-field interaction concept is introduced attain high porosity states, represent irregular shaped grains and incorporate the physical presence of bond fragments.
- Model is framed to be scalable and to include sample size effects.
- A coupled continuum-discrete approach is used to investigate model performance at the boundary value level.

## Introduction

Soft rocks may be classified as rocks with an Unconfined Compressive Strength (UCS) ranging from 0.5 to 25 MPa (Johnston 1991). This low UCS depends on their microstructure, result of sedimentation and diagenesis processes, characterized by bonded irregular-shaped grains (Ciantia et al. 2015b). Along with a low strength, these rocks also exhibit collapsible behaviour because of their high porous nature (Cuccovillo and Coop 1999). When stressed, the rock may crush and collapse developing irreversible deformations (Ciantia and Hueckel 2013) transforming the intact rock to a granular material. As shown by Lagioia and Nova (1995) from a constitutive modelling perspective, this class of materials have a rock type of behaviour that transitions to a soil like one once the structure is damaged because of loading. When sheared under low confinements localized dilatant brittle failure typical of rocks is observed (Leuthold et al. 2021). On the other hand, if the confinement pressure is high enough, when sheared the rock shows two competing effects: (i) softening because of bond damage and (ii) hardening induced by the evolving confined granular structure. These complex features are the main reasons why the design of displacement piles in soft rocks is challenging (Doghman et al. 2020) and often based on conservative empirical methods (Lord et al. 2002). For this reason, in the last decade, with the aim of improving foundation design in this peculiar material behaviour, extensive field-testing campaigns and physical modelling tests of displacement piles in this class of geomaterials has been performed (Alvarez-Borges et al. 2018; Jardine et al. 2023; Riccio et al. 2023). Key finding from these test results is the crushing and collapse of the rock during installation.

From a numerical perspective, simulating pile installation in such a material is extremely challenging. Recent development in advanced numerical methods such as the Smoothed Particle Hydrodynamics [SPH, see, e.g., Bui et al. (2011)], the Material Point Method [MPM, see, e.g. Soga et al. (2016)] and the Particle Finite Element Method [PFEM, see, e.g., Monforte et al. (2022)] has allowed to investigate in a rational and accurate way the interpretation of complex deformation processes in the ground undergoing extreme deformations. Continuum methods, however, require an appropriate constitutive model and ultimately rely on its ability to capture the material behaviour. An alternative to these continuum-based methods is the discrete element method (DEM). Funded on simple physically based interparticle interactions, the DEM has shown to be a promising tool to investigate installation effects on the ground-structure interaction of various geotechnical engineering foundation systems (Arroyo et al. 2011; Cerfontaine et al. 2023). On top of being able to manage large deformations and soil structure interaction the DEM also offers micromechanical insights on material behaviour (Ciantia et al. 2019a). Nonetheless its application has been mainly related to granular system and only few studies have used the DEM to investigate the micromechanics of rock structure interaction problems. Starting from the seminal work of Potyondy and Cundall (2004) the DEM studies on bonded materials have mostly focused on the investigation of the elementary response of dense rocks, looking at crack propagation patterns and analysing their micromechanical origins [see e.g. Jiang et al. (2015)]. DEM studies of rock/structure interaction in the literature concentrate more on steel/concrete–rock interface behaviour. For example, Gutiérrez-Ch et al. (2021) used DEM to study socketed piles in rock. In this case, installation does not affect the rock as the pile is grouted in a pre-drilled hole. To date DEM studies of the installation effects of displacement piles in soft rocks are few. Recently Zhang and Fatahi (2021) calibrated the flat-joint contact model to simulate the installation of an open-ended pile in a pyroclastic rock. The results are promising but were not validated with pile installation experimental data and the contact model scalability was not investigated. Calibration was performed on cm scale samples while the simulation was performed at the meter scale. In models where crushing is present, using particle scaling may introduce artefacts if strength size-effects are not properly separated from geometric scaling in the formulation. This kind of artefact is clearly very visible in the results by Falagush et al. (2015), showing an unrealistic concentration of crushing events away from the cone due to the geometrical scaling applied. As shown by Ciantia et al. (2015a), to avoid these particle scaling effects, the breakage model should be formulated in a scalable manner explicitly including particle scaling in the formulation.

Another key element to model soft rocks using DEM is to match the extremely high porous structure (porosity > 50%) to properly capture the collapse mechanism and at the same time being able to reproduce the post collapse shearing response. If spherical particles are used, the maximum porosity achievable for a loose packing is of about 0.42 (Zhang et al. 2006). Any DEM bonded model with spherical particles matching the experimental porosity of soft rocks would lead to excessive collapse mechanisms (Zheng et al. 2022) as the void ratio of the granular material after bond breakage at 5 kPa of confinement ranges from 0.45 to 0.5 (Jones and Addis 1986; Liu et al. 2023; Lagioia and Nova 1995). To overcome this limitation two strategies are possible: (i) use non spherical grains bonded to each other or (ii) use far-filed interaction laws whilst using spherical particles (Hentz et al. 2004). Finally, to capture the transition from intact to completely remoulded state, a physically based bond contact model is required. As clearly shown by Jiang et al. (2015) through a single bond experimental test campaign, bond limit force is strongly dependent on combined loading and is well described by H–M–V failure envelopes. The experimental data also shows that bond post peak behaviour is not instantaneous but often characterised by a softening trend. These experimental results were used by the same authors to propose for the first time a failure envelop based DEM contact model for cemented materials (Jiang et al. 2014; Shen et al. 2016). The model was shown to be able capture well dense and strong rock elemental behaviour. Building on this failure envelop concept, Dattola et al. (2014) developed a contact law able to capture the collapsible behaviour of calcarenites. To attain high porosities, smaller spheres were used to bond larger ones. This approach captured well the initial collapse, but post collapse behaviour and terminal porosities were not investigated. More recently Nguyen et al. (2017, 2019) developed a new family of damage-based contact models able to capture very well the inception and propagation of cracks in cemented materials. The authors propose a damage law related to fracture energy, linking bond damage with plastic deformation, and successfully reproduce compressive dilation at the particle scale. To replicate the behaviour of sandstone and Brisbane tuff Senanayake et al. (2022) extended these models introducing a cap to the failure envelop hence accounting for failure modes in compression at the contact scale.

In this work, a new contact model able to overcome the difficulties related to modelling cemented highly porous geomaterials with the DEM is proposed. As the final goal is to simulate boundary value problems (BVPs) such as pile installation, the contact model aims for (i) efficiency, (ii) ability to describe the collapsible behaviour of soft rocks and (iii) the capability of capturing the transition from the intact bonded state to a completely remoulded (granular) one. Based on the macro-element framework (Nova and Montrasio 1991), the contact model is developed to capture bond damage under compression, tension, shear and bending. The complexity of the model is compensated using far-field interaction as the broken bond is not physically modelled with smaller bonded fragments and present real physical interaction of irregular particles. It is shown how model parameters can be calibrated to match the mechanical behaviour of a soft rock at various confinement pressures through a series of element tests. A calibration procedure for representative soft rocks is proposed. To further decrease the computational burden for BVP simulations a coupled discrete-continuum approach is used (Li et al. 2022; Song et al.; 2022; Zheng et al. 2022). Here a 3D coupled DEM-FDM (finite differential method) approach is employed and the ability of the calibrated model to cope with complex BVPs is demonstrated by simulating the installation of a cone-ended model pile in Maastricht calcarenite.

## Novel Damage-Plasticity Bond Model

### Contact Law

**Fig. 1:**
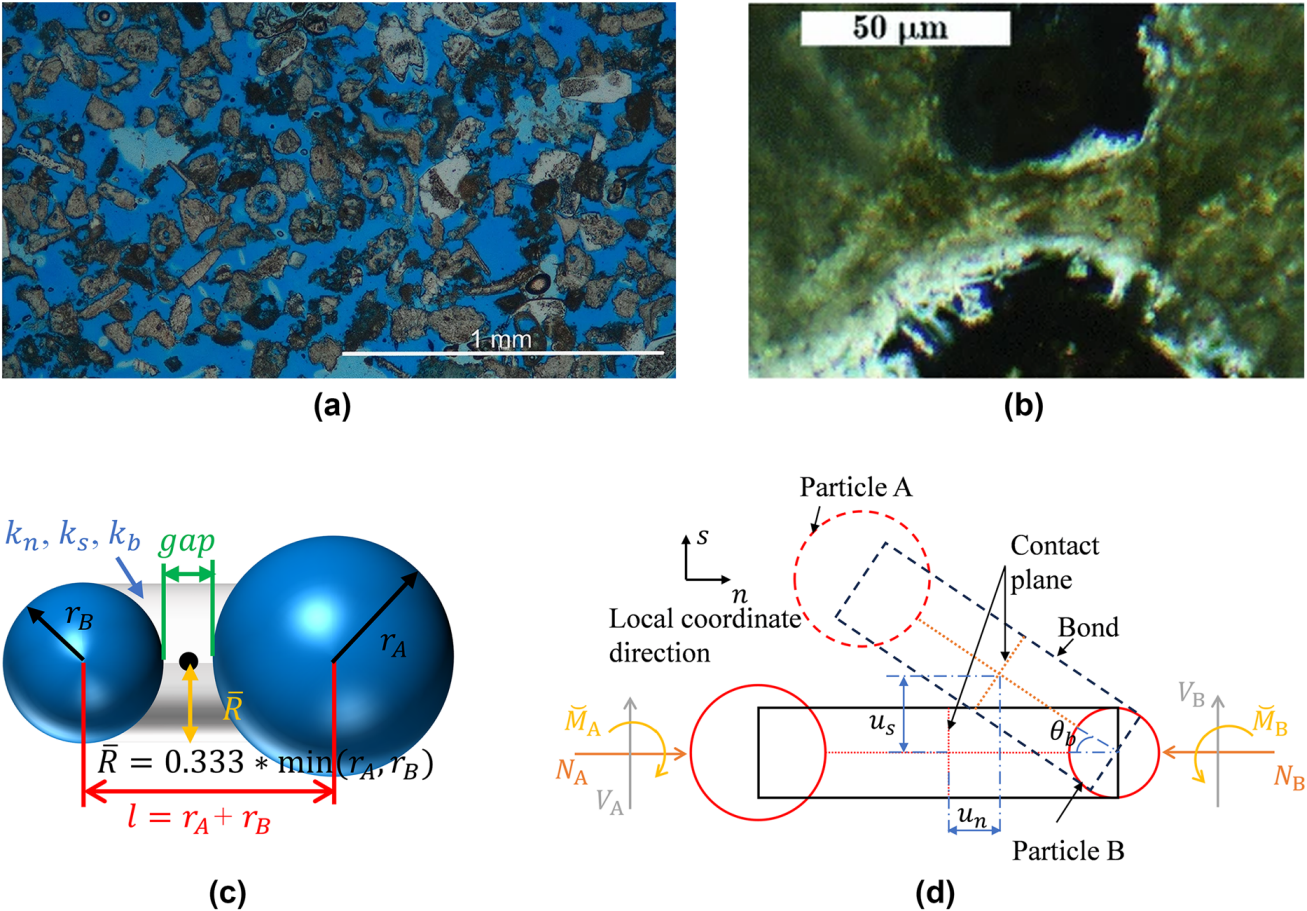
**a** Thin section of Maastricht calcarenite (Leuthold et al. 2021), **b** detail of two calcite grains bonded together in Calcarenite (Ciantia et al. 2015b) **c** geometrical definitions of DEM bond model and **d** bond kinematic and force definitions

The microstructure of a typical soft rock, observed under an optical microscope and shown in Fig. 1a, is filled with irregular inter-and intra-particle pores (blue region) (Leuthold et al. 2021) and Fig. 1b shows a typical bond of porous cemented soft rocks (Ciantia et al. 2015b). Based on such experimental evidence the characteristic dimensions along with kinematic quantities describing the motion of the proposed DEM bonded model are reported in Fig. 1c, d respectively. Most DEM models typically take the bond radius to be the size of the particle size, as these models mainly relate to dense and hard rocks (Brown et al. 2014; Potyondy and Cundall 2004; Zhang et al. 2023). However, in highly porous soft rocks, to better reflect the observed microstructure a smaller factor is necessary. In this work a factor of 1/3 as indicated in Fig. 1c is employed.

For load combinations below the yield limit, bonds will undergo elastic deformation based on Hooke’s law. Beyond the yield limit, irreversible bond displacements and rotation will develop. ${u}_{{\mathrm{n}}}$ and ${u}_{{\mathrm{s}}}$ (the normal and shear displacement of the bond, respectively) and ${\theta }_{{\mathrm{b}}}$ (the relative bond rotation) are subdivided into elastic and plastic components:

1 $$\left[\begin{array}{l}{u}_{{\mathrm{n}}}\\ {u}_{{\mathrm{s}}}\\ {\theta }_{{\mathrm{b}}}\end{array}\right]=\left[\begin{array}{l}{u}_{{\mathrm{n}}}^{e}\\ {u}_{{\mathrm{s}}}^{e}\\ {\theta }_{{\mathrm{b}}}^{e}\end{array}\right]+\left[\begin{array}{l}{u}_{{\mathrm{n}}}^{p}\\ {u}_{{\mathrm{s}}}^{p}\\ {\theta }_{{\mathrm{b}}}^{p}\end{array}\right]$$

The superscripts $e$ and $p$ correspond to the elastic and plastic (irreversible) parts, respectively. Figure 1d shows a schematic diagram of these three kinematic components. The forces and normalised bending moment (generalised loads) are related to the elastic bond displacement and expressed as

2 $$\left[ {\begin{array}{*{20}l} N \\ V \\ {\overset{\lower0.5em\hbox{$\smash{\scriptscriptstyle\smile}$}}{M} } \\ \end{array} } \right] = \left[ {\begin{array}{*{20}l} {k_{n} \left( {u_{n} - u_{n}^{p} } \right)A} \\ {k_{s} \left( {u_{s} - u_{s}^{p} } \right)A} \\ {k_{b} \left( {\theta _{b} - \theta _{b}^{p} } \right)/\bar{R}} \\ \end{array} } \right] = \left[ {\begin{array}{*{20}l} {\left( {1 - D} \right)k_{n}^{0} \left( {u_{n} - u_{n}^{p} } \right)A} \\ {\left( {1 - D} \right)k_{s}^{0} \left( {u_{s} - u_{s}^{p} } \right)A} \\ {\left( {1 - D} \right)k_{b}^{0} \left( {\theta _{b} - \theta _{b}^{p} } \right)/\bar{R}} \\ \end{array} } \right]$$

With $$N$$ and $$V$$ being the normal and shear forces on the bond; $$\overset{\lower0.5em\hbox{$\smash{\scriptscriptstyle\smile}$}}{M}$$ is the bending moment on the bond normalised by the bond radius $$\overline{R }$$; $$A$$ is the cross-sectional area of the bond; $$D$$ is an internal variable of the bond representing its level of damage, its value varies from 0 (intact) to 1 (broken); $${k}_{{\mathrm{n}}}^{0}$$ and $${k}_{{\mathrm{s}}}^{0}$$ are the initial normal and shear stiffness; $${k}_{{\mathrm{b}}}^{0}$$ is the initial bending stiffness, $${k}_{{\mathrm{b}}}^{0}={k}_{{\mathrm{n}}}^{0}I$$, $$I$$ is the inertia moment of the bond. Following Nguyen et al. (2017) bond damage evolution can be framed as:

3 $$D=1-{{\mathrm{exp}}}^{-\left(\frac{\left|{u}_{{\mathrm{n}}}^{p}\right|}{{u}^{c}}+\frac{{u}_{{\mathrm{s}}}^{p}}{{u}^{c}}+\frac{{\theta }_{{\mathrm{b}}}^{p}}{{\theta }^{c}}\right)}$$

${u}^{c}$ and ${\theta }^{c}$ are parameters that control the softening rate and are related to the fracture energies of the bond for the respective loading regime. The absolute value of the normal plastic displacement is used to account for damage in both tension and compression.

To avoid particle scaling effects on the elastic response of the bonded assembly Potyondy and Cundall (2004) introduced the effective modulus ${\overline{E} }_{{\mathrm{mod}}}$ and normal-to-shear stiffness ${\overline{\kappa }}^{*}$ expressed as

4 $$\begin{aligned} \overline{E}_{\bmod } & = \overline{k}_{{\mathrm{n}}} *l \\ \overline{\kappa }^{*} & = \frac{{\overline{k}_{{\mathrm{n}}} }}{{\overline{k}_{{\mathrm{s}}} }} \\ \end{aligned}$$

where ${\overline{k} }_{{\mathrm{n}}}$ and ${\overline{k} }_{{\mathrm{s}}}$ are the current normal and shear stiffness of the bond, $l$ is the bond length defined as the sum of the radii of the two particles connected by the bond.

#### Yield Surface

The bonds will behave in an elastic state if the generalised load lies within a field envelop. To describe the bond damage under combined loading, the yield surface $f$ accepted in Dattola et al. (2014)’s model is used. $f$ is expressed as

5 $$f={\left(\frac{\overset{\lower0.5em\hbox{$\smash{\scriptscriptstyle\smile}$}}{M}}{\widetilde{M}}\right)}^{1.001}+{\left(\frac{N}{\overline{N} }\right)}^{2}+{\left(\frac{V}{\overline{V} }\right)}^{4}{\left[1-{\left(\frac{N}{\overline{N} }\right)}^{2}\right]}^{-1}-1$$

The size of the fielding domain is controlled by the bond strength parameters $\widetilde{M}, \overline{N }$ and $\overline{V }$ that decrease linearly with the level of bond damage $D$:

6 $$\left[ {\begin{array}{*{20}l} {\bar{N}} \\ {\bar{V}} \\ {\tilde{M}} \\ \end{array} } \right] = \left[ {\begin{array}{*{20}l} {\bar{N}} \\ {\bar{V}} \\ {{\raise0.7ex\hbox{${\bar{M}}$} \!\mathord{\left/ {\vphantom {{\bar{M}} {\bar{R}}}}\right.\kern-\nulldelimiterspace} \!\lower0.7ex\hbox{${\bar{R}}$}}} \\ \end{array} } \right] = \left[ {\begin{array}{*{20}l} {\sigma _{0} A} \\ {CA} \\ {\sigma _{{\mathrm{t}}} {\raise0.7ex\hbox{$I$} \!\mathord{\left/ {\vphantom {I {\bar{R}^{2} }}}\right.\kern-\nulldelimiterspace} \!\lower0.7ex\hbox{${\bar{R}^{2} }$}}} \\ \end{array} } \right]\left( {1 - D} \right)$$

$${\sigma }_{0}$$ and $$C$$ are the normal and shear strengths, respectively. $${\sigma }_{0}={\sigma }_{{\mathrm{t}}}$$ under tension and $${\sigma }_{0}={\sigma }_{{\mathrm{c}}}$$ under compression, $${\sigma }_{{\mathrm{t}}}$$ and $${\sigma }_{{\mathrm{c}}}$$ are the tensile and compressive strength of the bond. The 3D yield surface hence gradually shrinks with increasing bond damage until the bond failure ($$D=1)$$.

As for classic strain hardening plasticity, whenever the generalised load on the bond wants to exceed the yield surface, plastic generalised bond displacements will develop to fulfil consistency conditions. In this work an associated flow rule is used and therefore the plastic potential $g=f$. The plastic deformation increment ($\delta {u}_{{\mathrm{n}}}^{p}, {\delta u}_{{\mathrm{s}}}^{p}, \delta {\theta }_{{\mathrm{b}}}^{p}$) can be hence expressed as

7 $$\left[\begin{array}{l}\delta {u}_{{\mathrm{n}}}^{p}\\ {\delta u}_{{\mathrm{s}}}^{p}\\ \delta {\theta }_{{\mathrm{b}}}^{p}\end{array}\right]=\varLambda \left[\begin{array}{l}\frac{\partial g}{\partial N}\\ \frac{\partial g}{\partial V}\\ \frac{\partial g}{\partial \overset{\lower0.5em\hbox{$\smash{\scriptscriptstyle\smile}$}}{M}}\end{array}\right]$$

where $\varLambda$ is the plastic multiplier. “Appendix 1” section summarises the integration scheme used and the expression of $\varLambda$. “Appendix 2” section reports the algorithm used to identify the coordinates of the closest point of the generalised load to the yield surface during a load increment of a bond in plastic sate.

#### Far-Field Interaction

As mentioned above, using spherical particles to match the initial extremely high porous structure of soft rocks and concurrently capturing the collapse mechanism until the bonded rock turns to a completely remoulded state is not possible. The maximum porosity achievable for spherical packing is 0.42 (Zhang et al. 2006) while remoulded soft carbonate rocks at 5 kPa of confinement have porosities greater than 0.45 (Jones and Addis 1986; Liu et al. 2023; Lagioia and Nova 1995). To overcome this limitation, following Hentz et al. (2004) the far-filed interaction law is used. This is clearly a model simplification but allows to exploit the efficiency of using spherical particles.

**Fig. 2:**
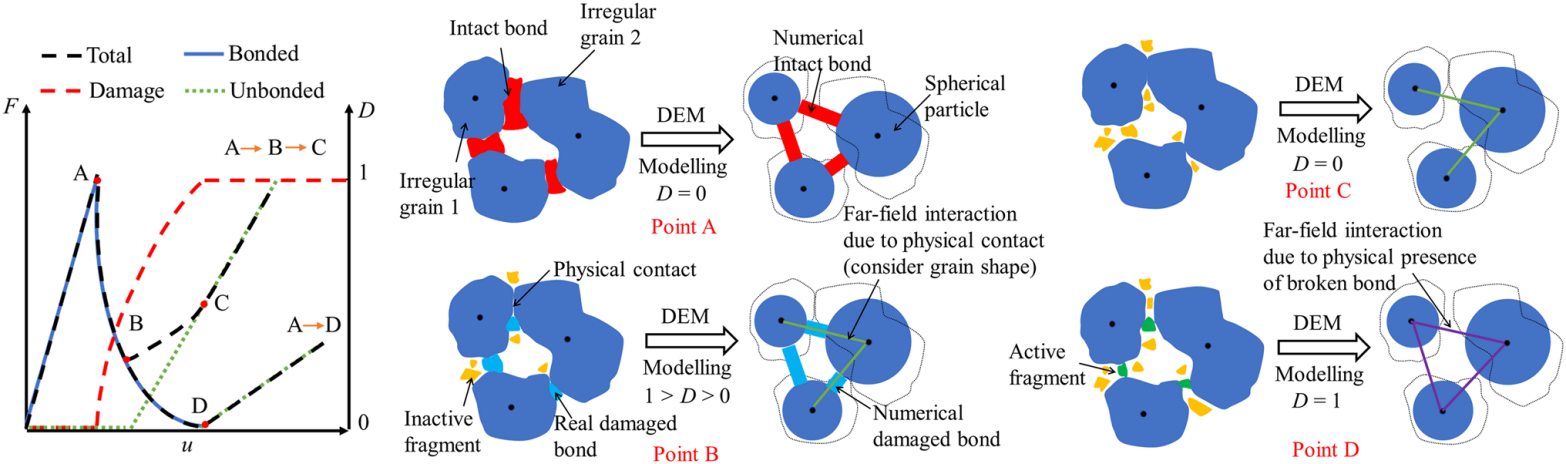
Schematic diagram outlining main features introduced by the far-field interaction concept

A physical justification of such choice is represented in the schematic of Fig. 2 that shows the force transmission between two bonded grains during the damaging process of a generic highly porous rock. Upon loading the bond between grain 1 and 2 behaves elastically until yield limit is reached (point A). At this point, a debonding process starts. As evinced by micro-experiments (Ciantia et al. 2015c), after the peak, some bond fragments detach from the intact bond, plastic displacements evolve and eventually grain–grain contact between two particles is attained (A-B). Before the grains go into contact (point D), the emergence of purely frictional grain-grain interaction forces is still feasible due to the physical presence of bond fragments. By means of a far-field interaction approach, aiming for computational efficiency, bond fragments are not explicitly represented in the simulations, but their presence is accounted for. As shown in the schematic of point B a far-field interaction approach allows to account for contacts between irregular shaped particles whilst using spheres. In terms of force transmission, in phase B–C the total force transmitted will be the sum of the remaining damaged bond and the frictional part from the physical contacts and fragments. Once the bond is completely broken the force transmitted will be governed by a typical frictional law here calculated using a purely frictional linear contact model defined by two elastic parameters (the effective modulus ${E}_{{\mathrm{mod}}}$ and normal-to-shear stiffness ${\kappa }^{*}$ of the particle) and a frictional one (interparticle friction coefficient $\mu$). From a modelling perspective such far-field interaction requires the introduction of an extra model parameter indicated as ‘activation gap ${g}_{{\mathrm{a}}}$’.

It should be noted that in this model, particle rotation is restricted if all neighbouring contacts are unbonded. This setting addresses the reduced interlocking forces between grains when using spherical particles (Arroyo et al. 2011). The assumption is reasonable because the fragments from broken bonds, filling the spaces between grains, help to prevent rotation. It is worth noting that the developed bond contact model can also be used to bond non-spherical particles modelled using either rigid blocks or clumps (Ye et al. 2019). In this case, at the cost of higher computational burden, fixing particle rotation would not be required (Ali et al. 2024).

#### One-Bond Contact Verification

As outlined in Table 1, to use this new contact model, a total of 8 parameters are required. Two control the elastic response of the bond (${\overline{E} }_{{\mathrm{mod}}}, {\overline{\kappa }}^{*})$, three (2 independent) are related to the strength of the bond (${\sigma }_{{\mathrm{t}}}, {\sigma }_{{\mathrm{c}}}, C$) and two control the softening rates (${u}^{c}, {\theta }^{c}$). The last two parameters are the activation gap of far-field interaction ${g}_{{\mathrm{a}}}$ and bond gap ${g}_{{\mathrm{b}}}$. These two parameters denote the distance at which bonds and far-field interactions are formed between untouched grains, respectively. When the conditions for purely frictional particle interaction are attained, a linear contact law governed by two elastic parameters (${E}_{{\mathrm{mod}}}, {\kappa }^{*}$) and interparticle friction coefficient $\mu$ is activated.

**Table 1:** DEM model parameters for one-bond contact verification and for Maastricht calcarenite

Parameters	Contact verification	Maastricht calcarenite
*Bond contact model*		
$\overline{E}_{{\mathrm{mod}}}$	400 MPa	22.5 GPa
$\overline{\kappa }^{*}$	2	2.4
$\sigma_{{\mathrm{t}}}$	1 MPa	36 MPa
$\sigma_{{\mathrm{c}}}$	10 MPa	198 MPa
$C$	1.5 MPa	40.5 MPa
$u^{c}$	0.1 mm	0.061 $d_{00}$ mm
$\theta^{c}$	0.1 mrad	0.04 rad
$g_{{\mathrm{a}}}$	(1.5 mm, 1 mm, 0)	0.15*d*_00_ mm
$g_{{\mathrm{b}}}$	0.01 mm	0.4 $d_{00}$ mm
*Linear contact model*		
$E_{{\mathrm{mod}}}$	400 MPa	0.63 GPa
$\kappa^{*}$	2	2.4
$\mu$	0.5	0.3

**Fig. 3:**
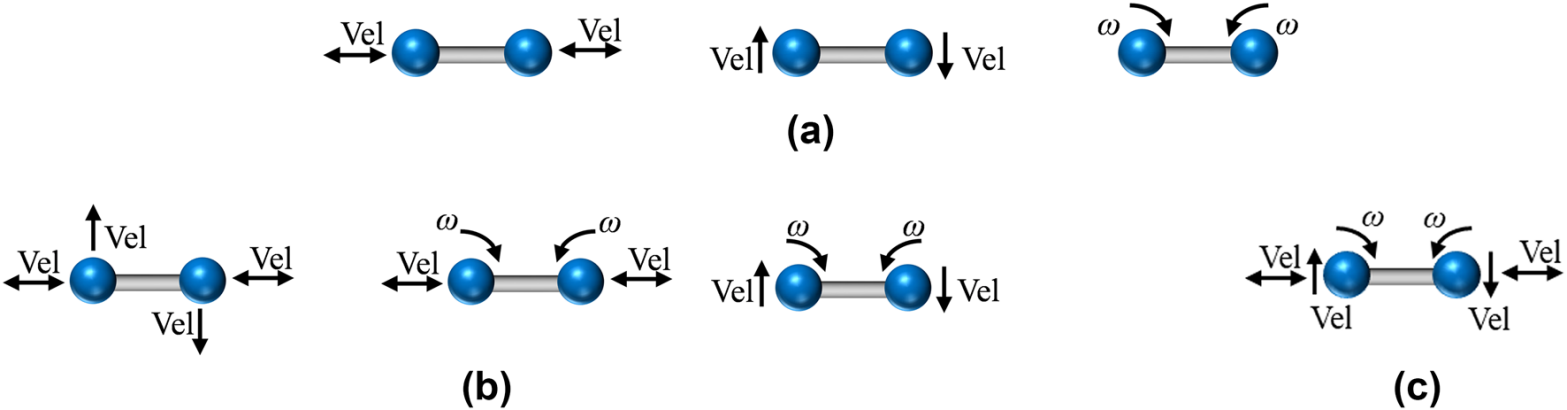
Loading modes considered for contact model verification: **a** one-loading mode; **b** two-loading mode and **c** three-loading mode

The model is implemented into the commercial software PFC 3D in the form of a user-defined model (Itasca Consulting Group 2018b). Refer to “Appendix 1” section for details related to the numerical integration and implementation. In this section, the performance of the new bond damage model is demonstrated by presenting the mechanical response of a one-bond contact under one, two and three loads, as shown in Fig. 3. The minimum radius of the two particles is 30 mm (i.e., bond radius $\overline{R }$ is 10 mm), and the other model parameters are summarized in Table 1.

**Fig. 4:**
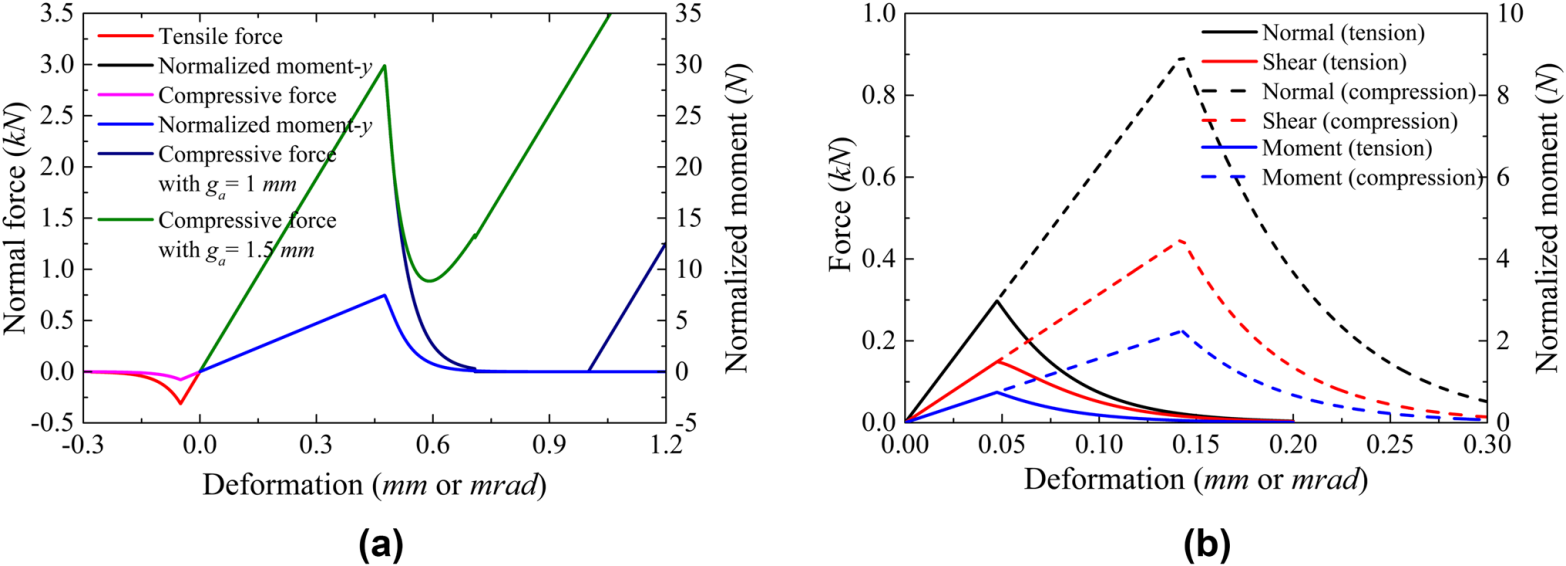
Generalised load–displacement relationship of the proposed contact model under **a** tension (compression)-rotation loading and **b** three-loading mode. In **a** the influence of $g_{{\mathrm{a}}}$ on the post peak behaviour is also reported

**Fig. 5:**
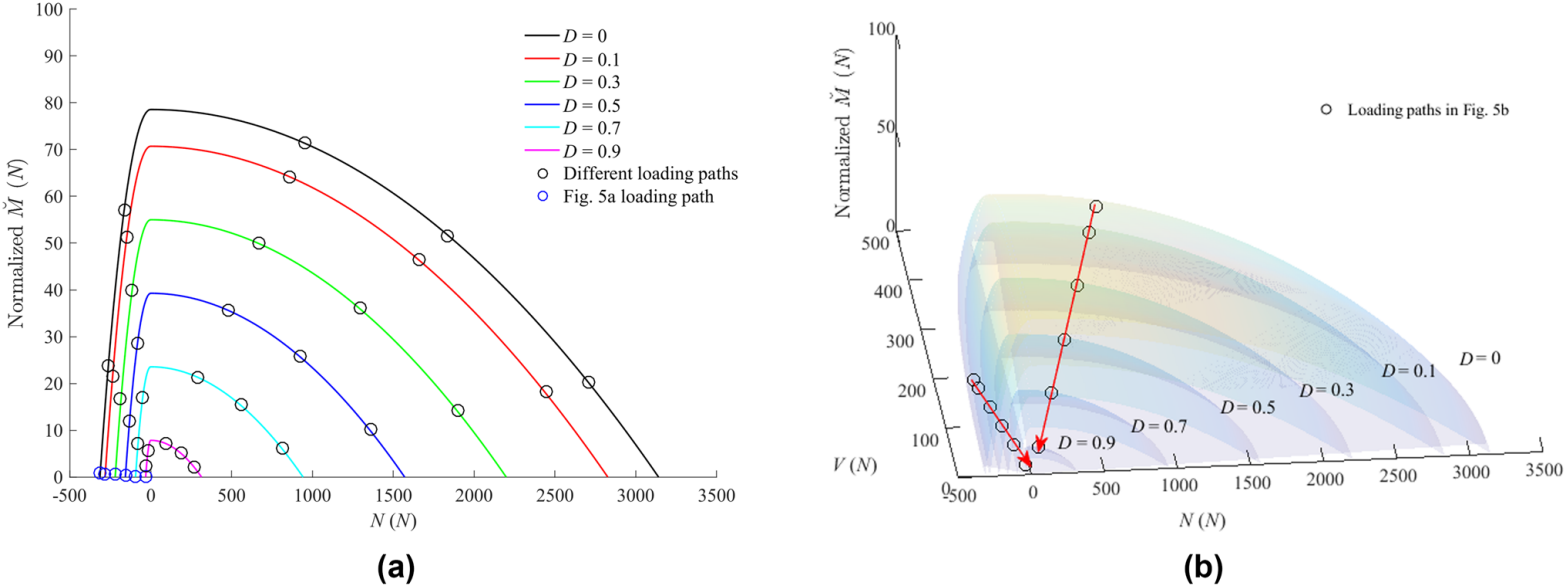
Evolution of the generalised load with respect to the yield surface shrinkage under **a** tension (compression)-rotation loading and **b** three-loading mode

To avoid clutter, only the results of tension (compression)-rotation and tension (compression)-shear-rotation loading modes are presented below. Figures 4 and 5, respectively, show the relationship between the generalised loads and deformations on the bond during loading, as well as the corresponding shrinkage of the yield surface with bond damage. Under the action of combined loads, the generalised loads on the bond decrease gradually with the development of bond damage. Figure 4a also illustrates the influence of ${g}_{{\mathrm{a}}}$ (far field interaction parameter) on the timing of compressive force recovery. Figure 5 presents such changes for different loading paths. In the figure it is shown how the updated generalized loads return to the evolving yield surface. This behaviour highlights the accuracy of the proposed algorithm detailed in “Appendix 2” section.

### Parametric Study

**Fig. 6:**
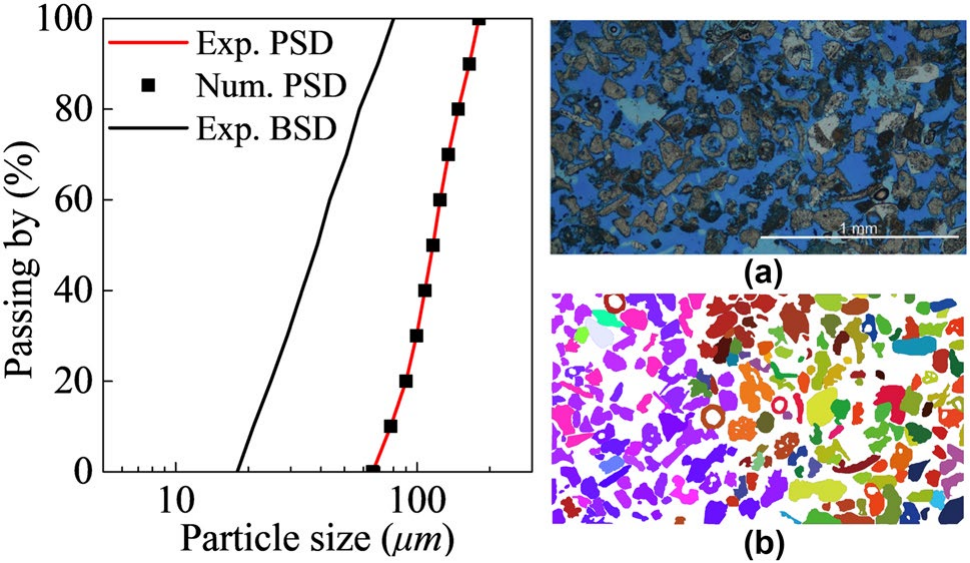
Particle size distribution (PSD) and bond size distribution (BSD) of Maastricht calcarenite obtain through image analysis of thin a section by (Leuthold et al. 2021)

For the parametric study a DEM cemented sample is generated using the radii expansion technique (Potyondy and Cundall 2004) in a 35 mm sided cube-shaped domain enclosed by periodic boundaries. Porous rock samples are generated with an initial porosity $n$ of 0.35 and 0.5 to represent typical dense and porous conditions, respectively. The employed upscaling ratio $S$ is 15 in this section. The dense and loose samples consist of approximately 14 k and 11 k particles with a particle size distribution (PSD) of Maastricht calcarenite (Fig. 6) obtained following Ciantia et al. (2015b) by image analyses of thin sections form Leuthold et al. (2021). As shown in Fig. 6b, 2D images were binarized through a saturation threshold and adjacent grains were separated using a watershed algorithm. PSD was determined calculating the equivalent circle radius for each grain. The bond size distribution (BSD), estimated using the length of the separation segments, is also represented in Fig. 6a. The initial porosity of Maastricht calcarenite is 0.52 with a median bond radius to median particle size ratio of 0.33. The dense sample is taken as an artificial material with the same bond to particle size ratio and a porosity of 0.35. In modelling bonded assemblies, a bond activation stage is always required. In this phase particles within a distance (see the gap in Fig. 1c) of each other (bond gap ${g}_{{\mathrm{b}}}$) are selected to be bonded. This length should be chosen such that the coordination number lies within physically sensible values observed in the lab. According to Fig. 1a, the coordination number of typical soft rocks should range between 3 and 5, which is also recommended by Jiang et al. (2013) and Kempton et al. (2012). For the dense and loose porous samples used here the coordination number is 5.0 and 3.6, respectively. In principle, the initial porosity of the model should be taken into account for the bond volume. In the literature this is rarely done. Herein for all the numerical samples considered, the bond volume was calculated and as it was found to be negligible (< 3% of the total particle volume) the same approach is used.

Table 2 summarises the combination of parameters for the parametric analysis performed to highlight the effects of strength and softening parameters, as well as the far-field interaction of the bond model on the macroscopic properties of the numerical sample. Because the compressive strength of soft rocks is greater than the tensile strength (Song et al. 2021), different ratios of ${\sigma }_{{\mathrm{c}}}$ to ${\sigma }_{{\mathrm{t}}}$ are investigated. Similarly, several different ratios of ${u}^{c}$ to ${\theta }^{c}$ are set to explore the effect of softening parameters on post-peak response.

**Table 2:** Variation of strength and softening parameters

Parameters	Unit	Reference value	Variation
$\sigma_{{\mathrm{t}}} \left( {\sigma_{{\mathrm{c}}} } \right)$	MPa	9 (90)	9 (180), 9 (450), 9 (900)
$C$	MPa	9	4.5, 18, 45
$\theta^{c} \left( {u^{c} } \right)$	Rad (mm)	10 (0.1)	2 (0.1), 5 (0.1), 20 (0.1)
$g_{{\mathrm{a}}}$	mm	0	0.01*d*_00_, 0.1*d*_00_, 0.2*d*_00_

$$E_{{\mathrm{mod}}} = 1\,{\mathrm{GPa}}$$, $$\overline{E}_{{\mathrm{mod}}} = 9\,{\mathrm{GPa}}$$ $$\kappa^{*} = { }\overline{\kappa }^{*} = 5$$, $$\mu = 0.5$$, $$g_{{\mathrm{b}}} =$$ 0.4*d*_00_ mm, *S* = 15

#### Effect of Strength Parameters on Elastic Domain

**Fig. 7:**
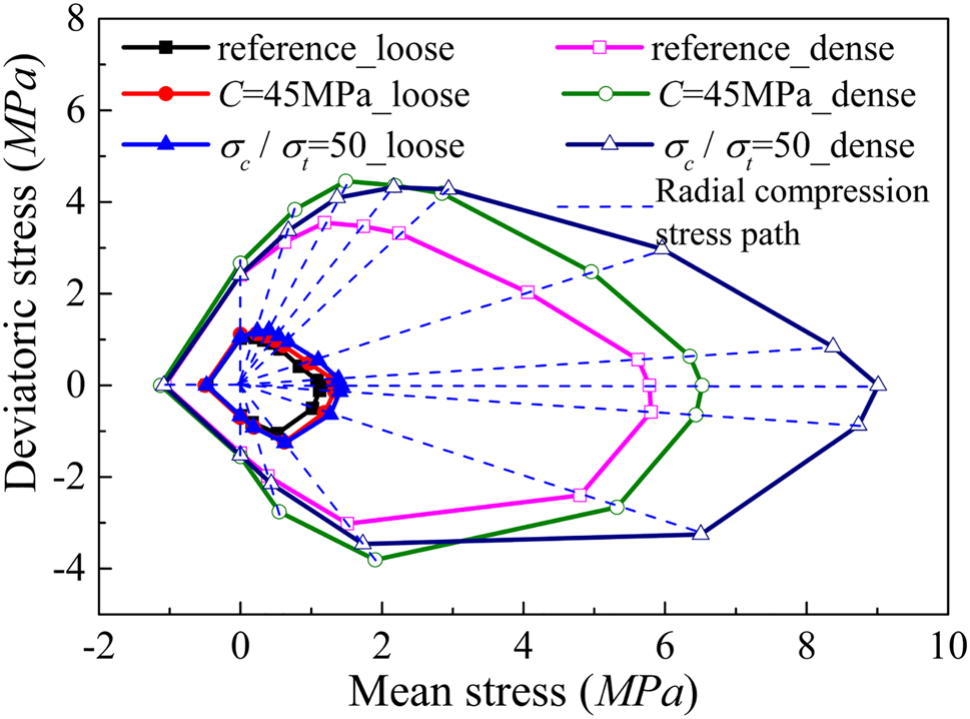
Elastic domains of dense and loose samples characterised by different combinations of bond strength parameters

To investigate the effect of strength parameters on the size of the yield surface, a series of simulations imposing different radial compression stress paths (∆*q*/∆*p*′ = constant) were conducted by imposing different stress paths. Figure 7 presents the elastic domains of dense and loose samples with varying strength parameters. The dense sample exhibits more significant variations when altering strength parameters and this is attributed to the higher coordination number.

#### Effect of Softening Parameters on Post-peak Response

**Fig. 8:**
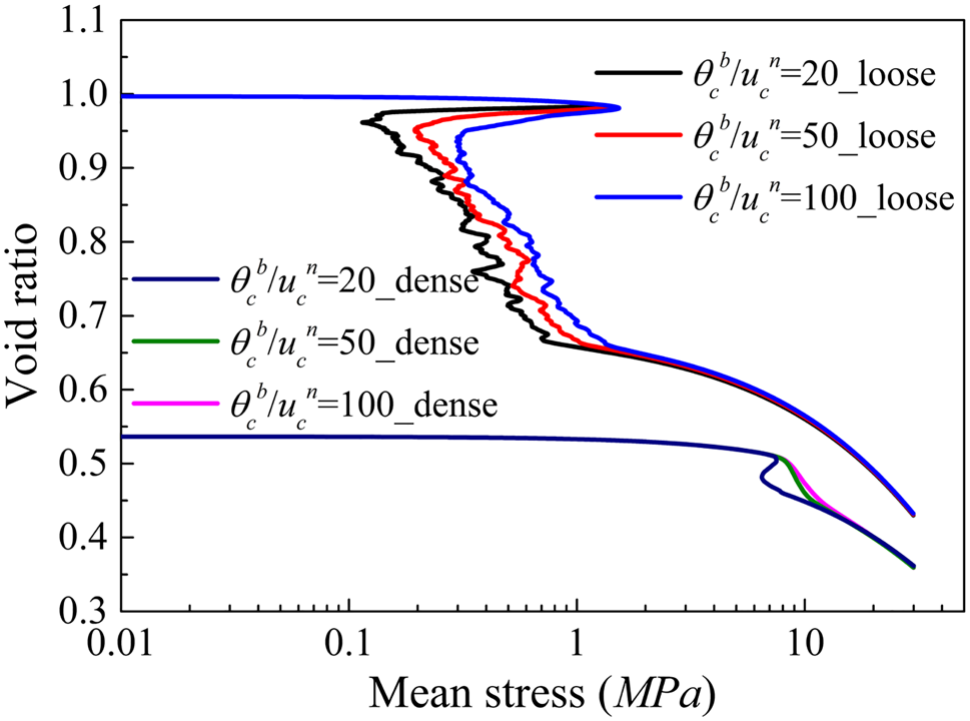
Isotropic compression response of dense and loose soft rocks for different sets of softening parameters

Figure 8 depicts the post-peak behaviour of dense and loose samples in isotropic tests. As expected, the brittleness is strongly affected by the softening parameter used. Interestingly, significant stress reductions are evident in the loose sample, even when using slow softening rates. Assigning a bond strength variability to the numerical sample can be used to avoid this abrupt collapse (Ciantia et al. 2014).

#### Effect of Interparticle Frictional Contact Activation on Postpeak Response

**Fig. 9:**
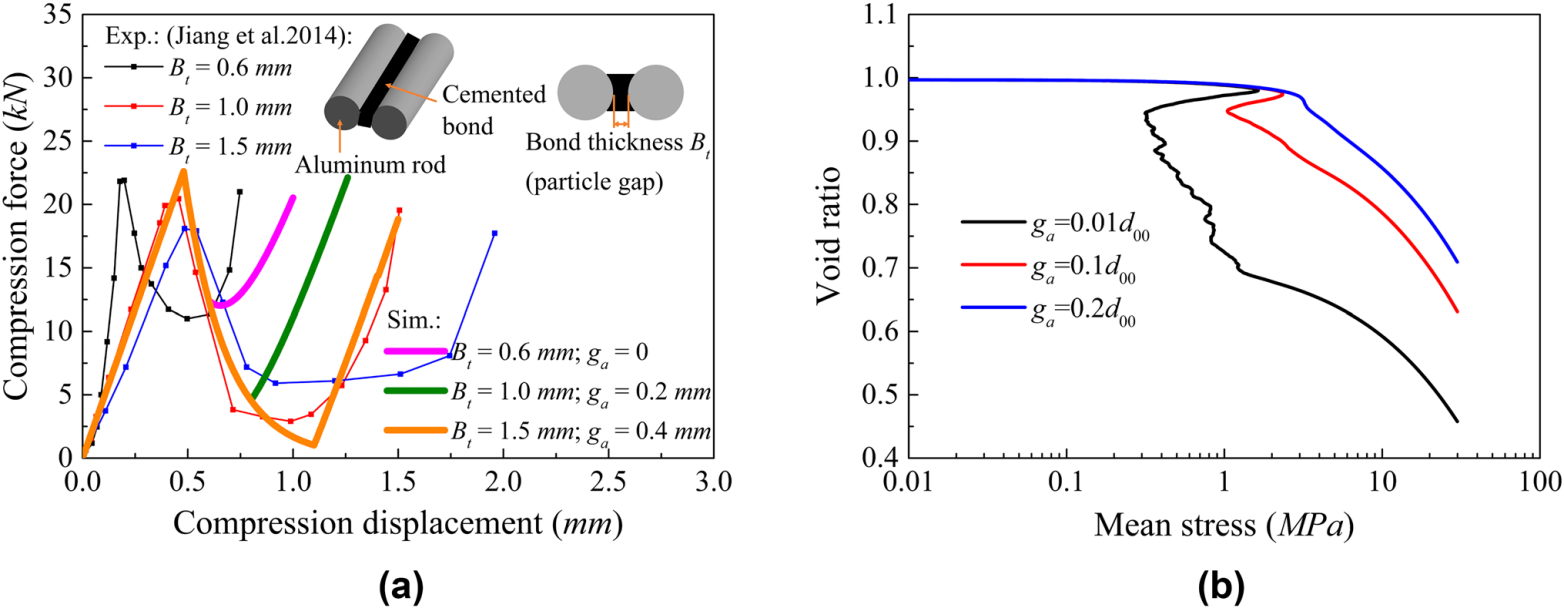
**a** Experimental results of idealised single bond response under different grain gaps (Jiang et al. 2014) and **b** effect of far-field interaction parameter on isotropic compression response

As outlined in Table 2, various values of ${g}_{{\mathrm{a}}}$ were used to investigate far-field interaction effects on the post-peak isotropic compression response of loose samples. As shown in Fig. 4a ${g}_{{\mathrm{a}}}$ controls the onset of the hardening response at contact level. A similar trend was also captured by Jiang et al. (2014), who conducted a conceptual one-bond compression tests demonstrating the effect of gap on the timing of recovery of contact force, see Fig. 9a. These trends are well reproduced by the proposed contact model. The parameters of the model for this fit are the same as those in the simulations shown in Fig. 4, except for ${\overline{E} }_{{\mathrm{mod}}}$ and ${E}_{{\mathrm{mod}}}$, both set to 10 GPa to capture the bond stiffness of the experiments. Based on the observed numerical and experimental behaviours at the contact level, Fig. 9b shows how ${g}_{{\mathrm{a}}}$ influences the compressive response at the macroscale, allowing to better reproduce experimental trends.

### Particle Upscaling Law

Upscaling particle size is often used to decrease the computation burden of DEM models (Ciantia et al. 2015a; Coetzee 2019). Fewer particles are used to fill the same volume by scaling their size by a factor *S*. This should not be confused with sample size effects whereby changes in rock sample size have a significant effect on the mechanical behaviour. While the elastic parameters of the model are already set to be scalable according to Eq. (4), strength parameters need to be scale independent. Zheng et al. (2022) show that the bond strength parameters (${\sigma }_{{\mathrm{t}}}, {\sigma }_{{\mathrm{c}}}$) are scale independent while post peak response is affected if the softening rates (${u}^{c}, {\theta }^{c}$) are not scaled. As Liu et al. (2018) show that softening is insensitive to particle size, here a linear upscaling law for ${u}^{c}$ is proposed while the rotation softening parameter ${\theta }^{c}$, being a dimensionless variable, is scale independent. Finally, also the bond activation gap will need to be scaled with a linear law. It therefore results that:

8 $$\left\{\begin{array}{l}{\overline{E} }_{{\mathrm{mod}},S}={\overline{E} }_{{\mathrm{mod}}}\\ {{\overline{\kappa }}_{S}}^{*}={\overline{\kappa }}^{*}\\ {\sigma }_{t,S}={\sigma }_{{\mathrm{t}}}\\ {\sigma }_{c,S}={\sigma }_{{\mathrm{c}}}\\ {u}_{S}^{c}={Su}^{c}\\ {\theta }_{S}^{c}={\theta }^{c}\\ {g}_{a,S}=S{g}_{{\mathrm{a}}}\\ {g}_{b,S}=S{g}_{{\mathrm{b}}}\end{array}\right.$$

**Fig. 10:**
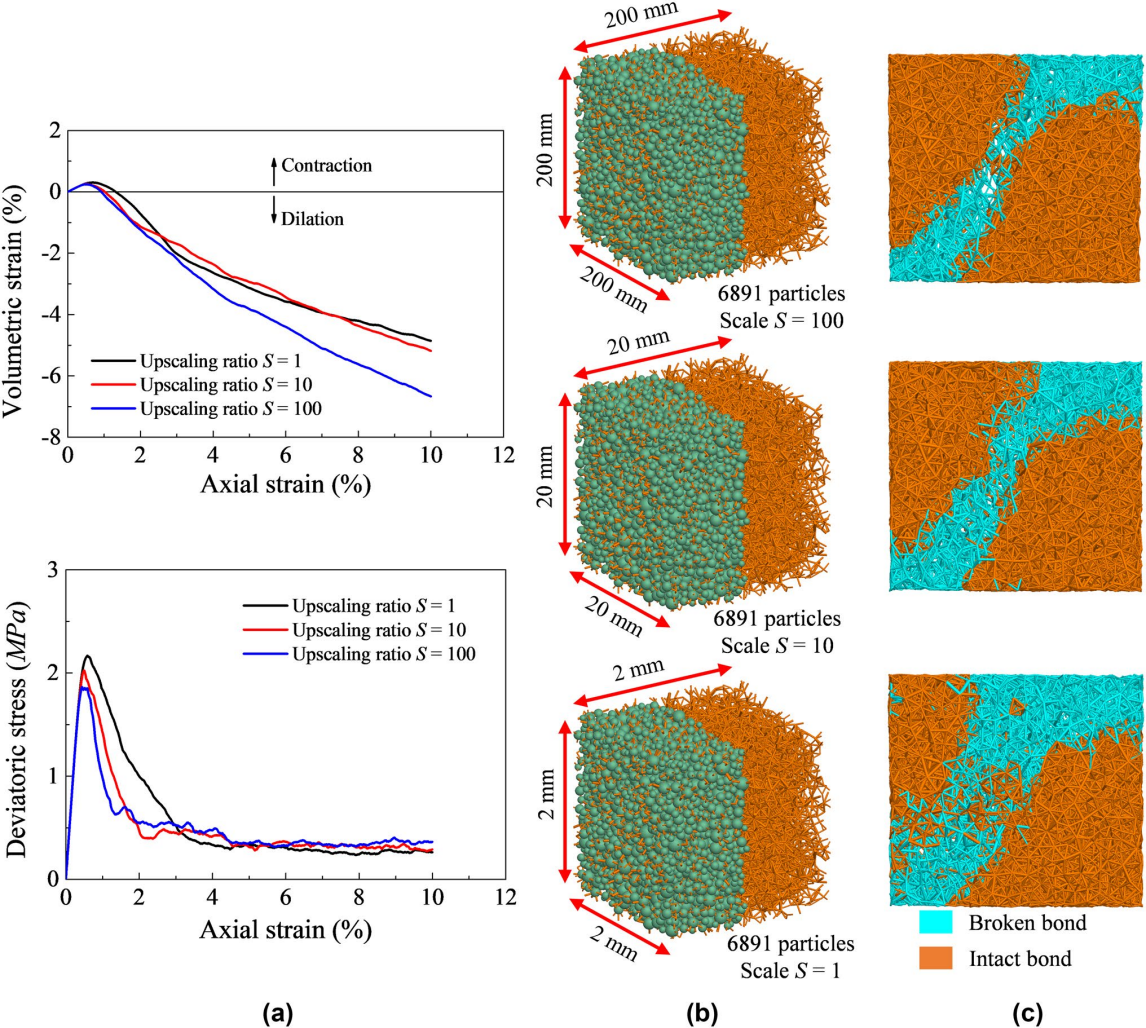
**a** Stress–strain macro response of triaxial compression under (100 kPa) confinement; **b** snapshot of DEM initial numerical samples and **c** snapshot of damaged and intact bonds at end of shearing showing similar response despite particle scaling

At this point, the model here proposed is framed to be scale independent and to demonstrate this, the mechanical response of three cubic numerical samples with different upscaling ratios *S* (see Table 3) is tested. Model elastic and strength parameters are the reference values listed in Table 2. The softening and the far-field contact parameters employed in this section are summarized in Table 3. As shown in Fig. 10, as long as the sample is large enough to be considered as a representative elementary volume (REV), the response is scale independent. Figure 10 provides scale independence evidence also from a micro scale viewpoint as similar failure modes caused by local shear bands are observed in all three samples.

**Table 3:** Softening and the far-field interaction parameters employed for upscaling validation

*S*	1	10	100
$d_{00}$ (mm)	6.6e−2	6.6e−1	6.6
$u^{c}$ (m)	1e−4	0.001	0.01
$\theta^{c}$ (rad)	1e−2	1e−2	1e−2
$g_{{\mathrm{a}}}$ (mm)	0.1 $d_{00}$	0.1 $d_{00}$	0.1 $d_{00}$

**Fig. 11:**
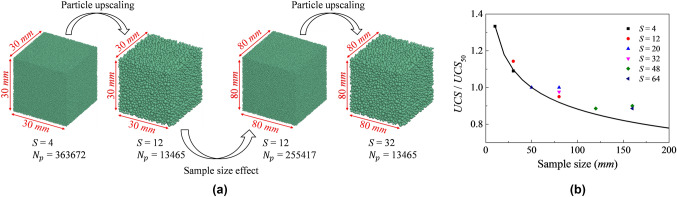
**a** Schematic diagram showing the difference between particle upscaling and sample size effects and; **b** influence of specimen size on the strength of intact rock with different particle upscaling ratios

Finally, the scalable contact model just presented can also be cast to incorporate size effects. Size effects are due to internal flaws like natural fissures whereby larger samples exhibit lower rock strength (Brown and Hoke 1980; Darlington et al. 2011; Masoumi et al. 2016; Shahin et al. 2019; Zhai et al. 2020). Size effects can be introduced in the contact model by assigning strength parameters as function of a reference sample size. Figure 11 shows how the new model here proposed can be modified to account for this whilst exploiting the power of scaling particle size. The elastic parameters correspond to the reference values provided in Table 2, while the strength and softening parameters are summarized in Table 5. The details of the size effect implementation can be found in “Appendix 3” section.

### Contact Model Calibration Procedure

**Fig. 12:**
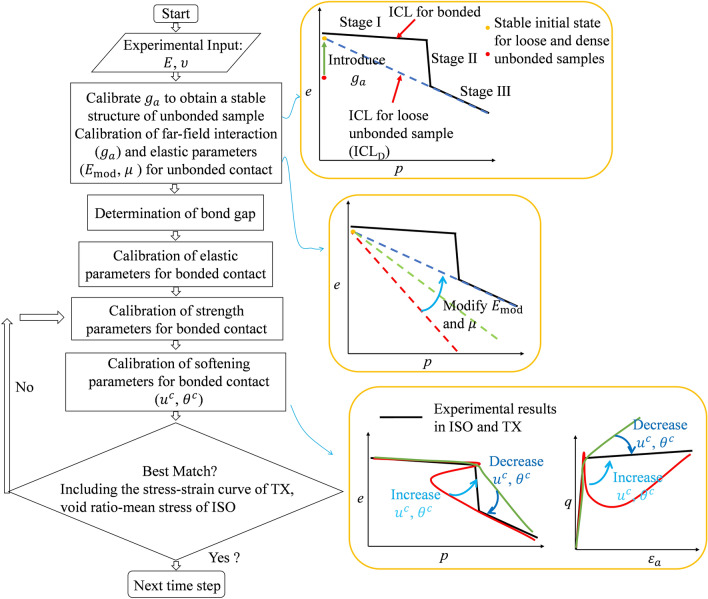
Flowchart outlining the contact model calibration procedure

The new DEM modelling framework requires 8 model parameters for the bond model and 3 for the destructured rock. To allow an efficient and simple use of it, a calibration procedure represented in the flowchart in Fig. 12 is proposed. As mentioned, high-porous soft rocks undergo three distinct stages during isotropic compression: an elastic response (stage I), a transition stage due to debonding (stage II) and finally a soil-like granular behaviour stage (stage III). Following the approach proposed by Lagioia and Nova (1995) by extending the ICL (Isotropic compression line) of the remoulded state backward, an ideal isotropic compression line (ICL_D_) for the unbonded sample can be identified. This can be used to determine the void ratio for the unbonded sample at its initial state. If the line crosses the intact ICL the point of intersection also indicates the preconsolidation pressure diagenesis and bonding took place. The following calibration procedure is a one-by-one matching process as each model parameter corresponds to a clear physical macroscopic property (Potyondy and Cundall 2004).

#### Step 1: Calibration of Far-Field Interaction and Elastic Parameters for Unbonded Contact

The Initial state (yellow point in Fig. 12) represents the microstructure of the destructured rock supported by irregular grains and broken bond fragments. To match this state, create a loose cloud of particles matching experimental PSD and equilibrate sample at 5 kPa of confinement. Identify the value of ${g}_{{\mathrm{a}}}$ to obtain a stable unbonded structure at the target initial porosity. Subsequently tune particle effective modulus ${E}_{{\mathrm{mod}}}$ and interparticle friction coefficient $\mu$ by matching the ICL in the isotropic test (ISO), see the schematic diagrams of the first two steps in the flowchart (Fig. 12).

#### Step 2: Determination of Bond Gap

Once *g*_*a*_ is calibrated, a cloud of particles matching experimental PSD and the initial porosity of the rock is generated. To match the initial porosity of the rock the volume of the bonds should be considered and therefore a few iterations are required. Where possible thin sections or SEM images of the microstructure should be used to calibrate the bond to particle size ratio. Activate bonds by setting a bond gap activation threshold *g*_*b*_ such that the coordination number is representative for the rock. The bond gap ${g}_{{\mathrm{b}}}$ is determined by matching the initial porosity and coordination number of the rock through analysis of CT images or thin sections experimentally, or by ensuring that it falls within the typical range of 3 to 5 for soft rocks in the absence of CT or X-ray measurements (Jiang et al. 2013; Kempton et al. 2012).

#### Step 3: Calibration of Elastic Parameters for Bonded Contact

Calibrate the elastic parameters ${\overline{E} }_{{\mathrm{mod}}}$ and ${\overline{\kappa }}^{*}$ (${\kappa }^{*}$) of the bond by matching the Young's modulus $E$ and Poisson's ratio $\upsilon$ through a triaxial simulation (TX).

#### Step 4: Calibration of Strength Parameters for Bonded Contact

The compressive strength ${\sigma }_{{\mathrm{c}}}$ should be calibrated using isotropic compression test simulations (to match the yield point in the ISO test), as bonds are primarily subjected to compressive forces. Triaxial simulations should then be used to calibrate ${\sigma }_{{\mathrm{t}}}$ and $C$, aiming to fit the shape of the experimental yield envelope. ${\sigma }_{{\mathrm{t}}}$ and $C$ are strongly related to each other and control the shape of the yield envelop at low confinements. A ratio of ${\sigma }_{{\mathrm{t}}}$/$C$ = 1 (corresponding to macroscopic low confinement peak friction angle of 45) is recommended. This can be slightly adjusted after calibration to better match the yield stress envelope at low confinement conditions through trial and error and its value should range between 0.8 and 1.2.

#### Step 5: Calibration of Softening Parameters for Bonded Contact

Softening parameters can be calibrated to match the post-peak behaviour of triaxial tests (Fig. 12). Another stress path (isotropic) is then used to validate the chosen parameters.

### Application of the Model to the Study of Maastricht Calcarenite

**Fig. 13:**
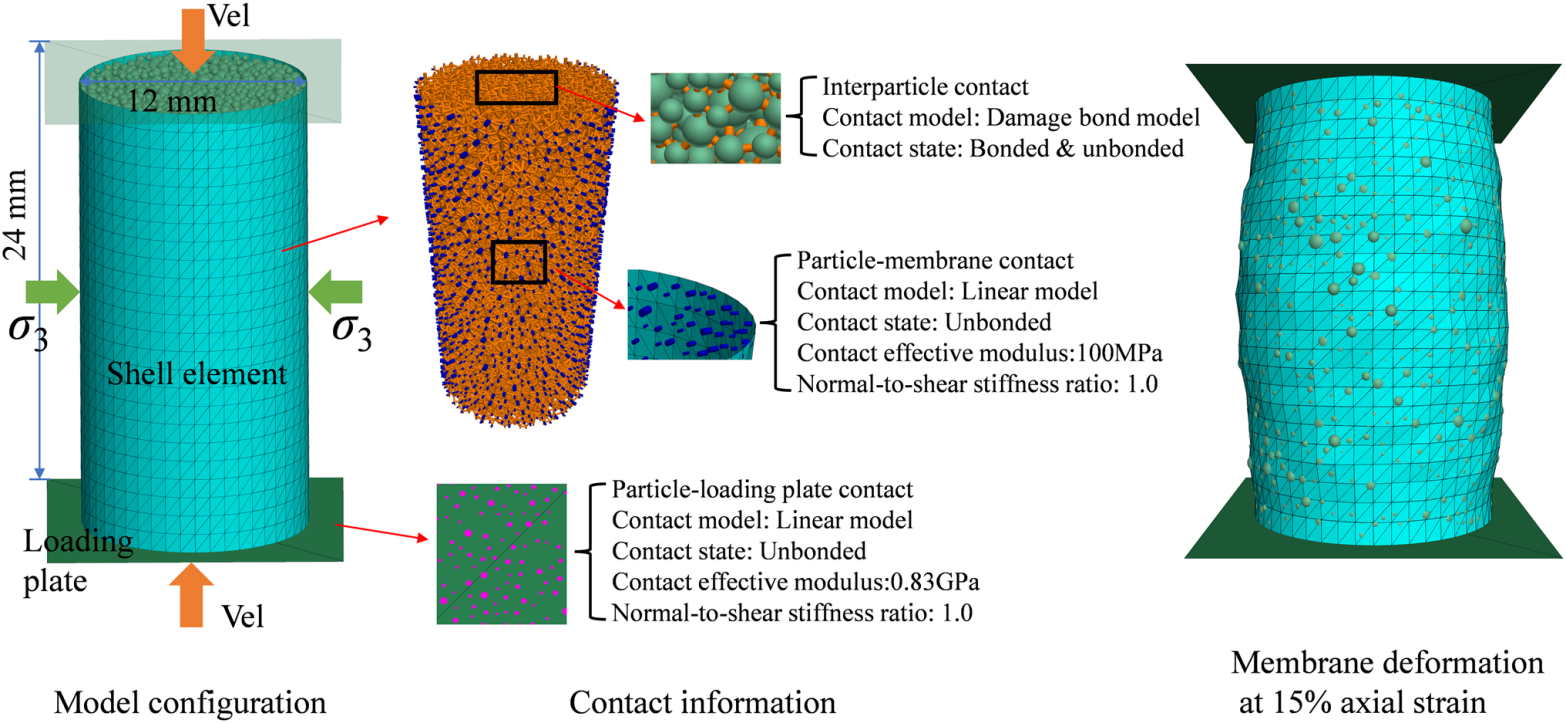
Configuration of coupled DEM-FDM triaxial simulation

**Fig. 14:**
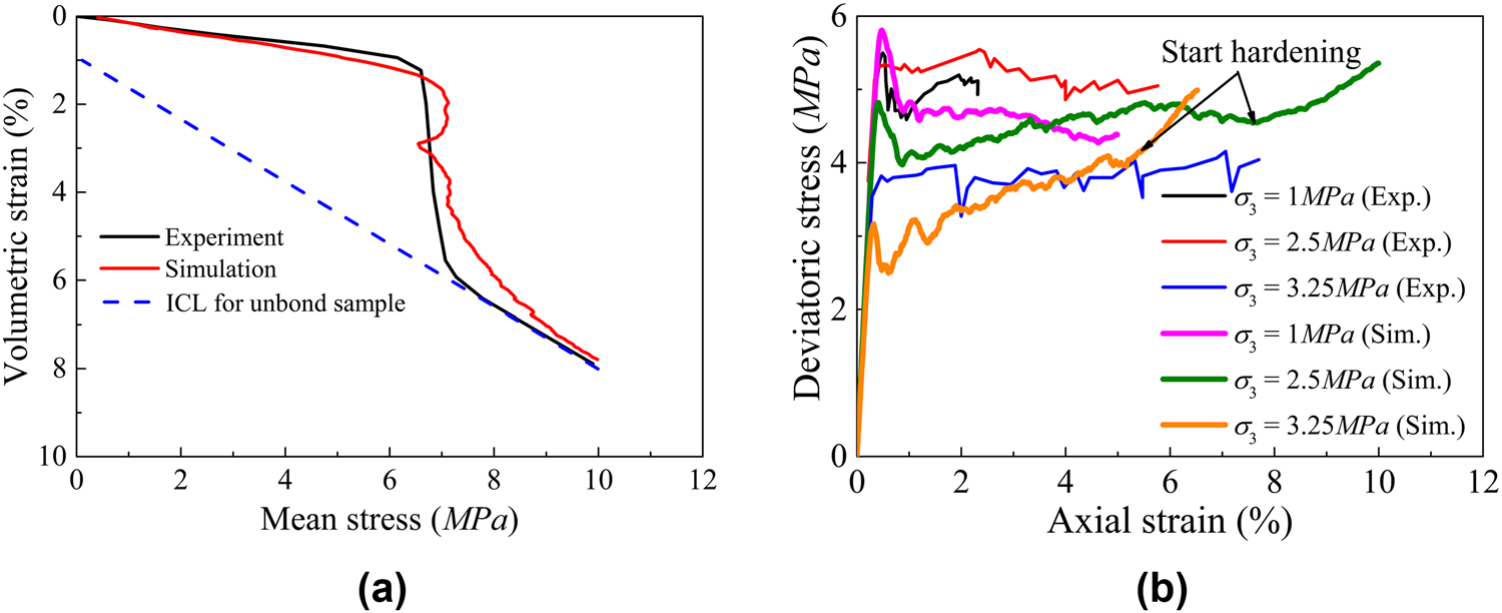
Calibrated model simulation of **a** isotropic compression and **b** triaxial compression

Maastricht calcarenite is a typical cemented soft rock with high porosity and low strength. It features an initial porosity $n$ of 0.52, a Poisson's ratio $\upsilon$ of 0.14, and a uniaxial compressive strength (UCS) of approximately 3.6 MPa (Leuthold et al. 2021). Following the procedure described in Sect. 2.4 a cylinder-shaped numerical sample as illustrated in Fig. 13 was prepared to perform isotropic and triaxial compression tests. The DEM model generated using an upscaling ratio $S=5$ comprises around 18 k particles, with dimensions of 24 mm in height and 12 mm in diameter. The coordination number in this sample was set to be 4 to fall between the experimental values of 3 and 5 according to Fig. 1a. All particles are enclosed by the cylinder-shaped membrane and rigid top and bottom plates. The flexible membrane is created using shell elements in another commercial software FLAC (Itasca Consulting Group 2018a) based on the FDM and has been validated to reproduce shear bands in triaxial simulations (Lv et al. 2023; Xiong and Wang 2023; Zhu et al. 2022). The load transfer between DEM particles and FDM shell elements is achieved through the coupled solution provided by Itasca Consulting Group (2018b), further details are reported in Xu et al. (2021). A linear contact model is applied between particles and the loading plate, as well as between particles and the membrane. To represent the real testing configuration in triaxial tests the effective modulus between particles and the steel loading plate is taken to be higher than that between particles and the membrane, and higher than the interparticle contact. The contact model was calibrated following the procedure above and the final calibrated parameters are reported Table 1. Figure 14a show the capability of the model to capture void collapse in isotropic compression while Fig. 14b the ability to predict the experimentally observed transition from softening to hardening when shearing the rock under both low and high confinements. An earlier hardening response is observed in the simulation under high confinements. This behaviour should be attributed to the overestimation of the far-field interaction effect, which caused by changes in the microstructure resulting from particle breakage and bond breakage.

**Fig. 15:**
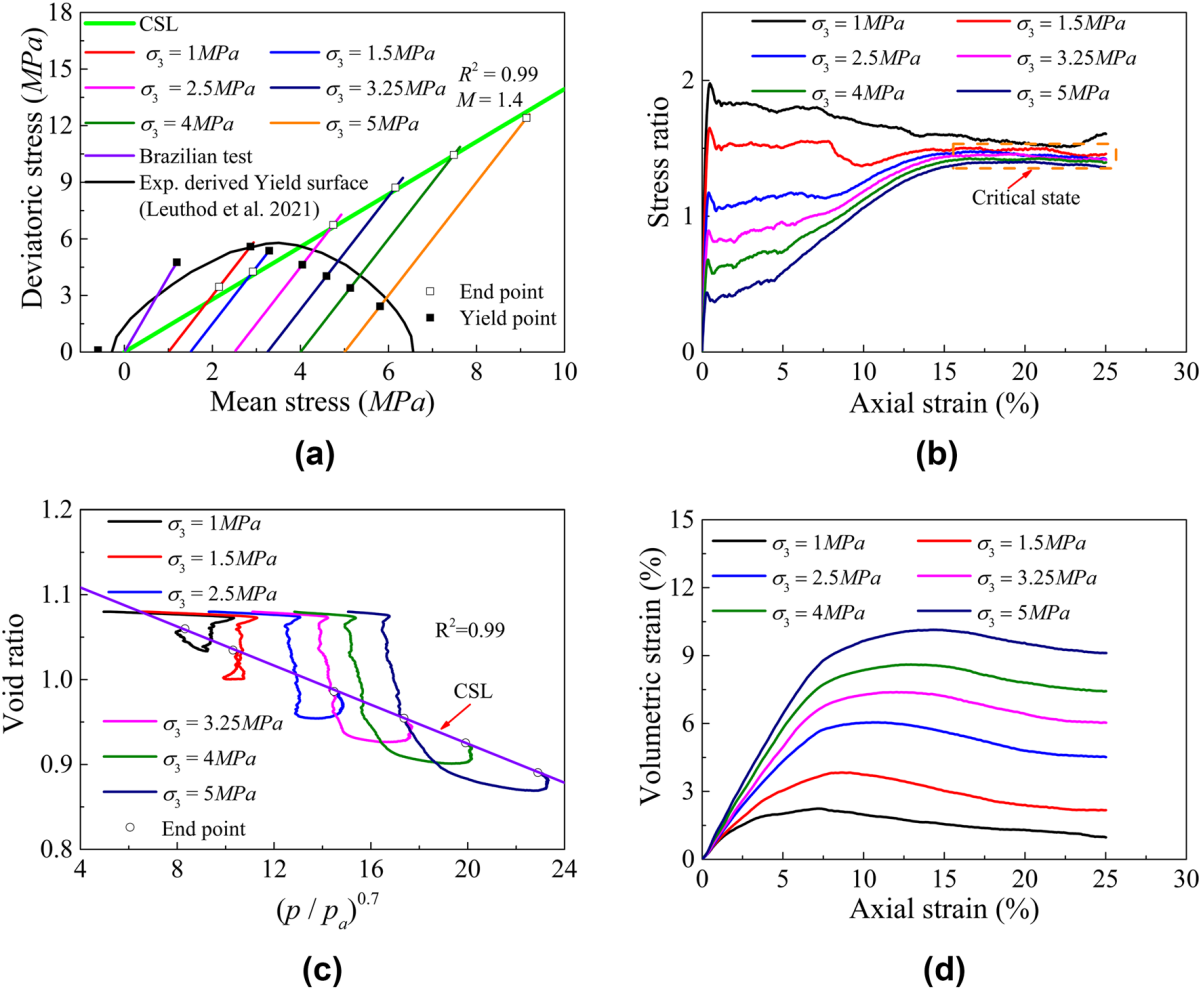
Numerical triaxial compression behaviour of the DEM model of Maastricht Calcarenite represented in the: **a** deviatoric stress-mean stress (q–p) plane; **b** stress-ratio axial strain plane; **c** e–p compression plane; **d** volumetric strain axial strain plane. In **a** the experimentally derived Yield surface from Leuthold et al. (2021) is also shown

**Fig. 16:**
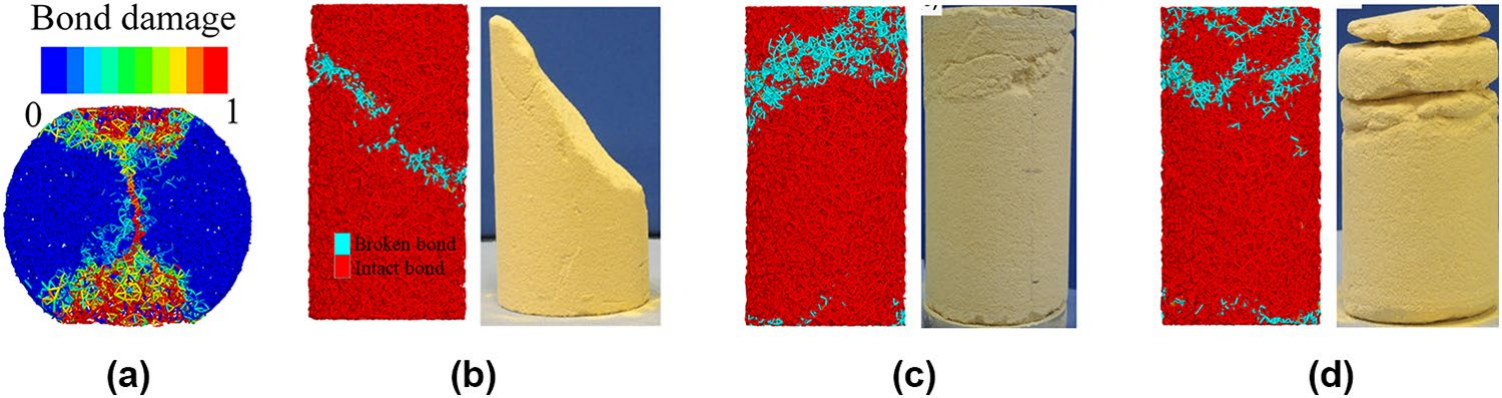
Bond damage distribution in **a** Brazilian and TX tests with varied confinements; **b** 1 MPa; **c** 1.5 MPa and **d** 2.5 MPa

To further evaluate model’s performance, a series of triaxial tests under different confinements are conducted to determine the yield points and critical state of this DEM model of Maastricht calcarenite. A comparison of the experimental yield surface line (YSL) with the numerical yield points is shown in Fig. 15. Several stress paths are also be plotted in Fig. 15a to highlight typical trends observed for low and high confined shearing tests. Figure 15b shows how the stress ratio tends to the same value once strains above 15% are attained. This corresponds to a macroscopic critical state friction angle of 34°. Note the stress ratio of the sample under low confinement (1 MPa) is not constant because an obvious shear band generated in the numerical sample (see Fig. 16b). It is worth noticing that even though samples under middle and high confinements reach a constant stress ratio after 15% of axial strain, the volumetric strain is still not constant (Fig. 15d). Figure 15c shows the linear CSL in the void ratio—$\left( {p/p_{a} } \right)^{{0.7}}$ plane, where ${p}_{{\mathrm{a}}}$ is atmospheric pressure (101 kPa). This linearity demonstrates the granular-type of behaviour of the remoulded rock also observed for crushable sands (Ciantia et al. 2019b).

To investigate the performance in predicting tensile failure, a Brazilian test is conducted using the calibrated model parameters and the same particle upscaling ratio. The sample size is 12 mm in diameter and 6 mm in height. Two rigid frictionless flat plates were used to load the sample. The bottom plate was fixed while the top one was displaced downwards at a fixed velocity of 0.1 cm/s until a peak lad was attained. As for the real experiment this value was used to calculate the tensile strength which in this case results equal to 2.2 MPa. As illustrated in Fig. 16a, all broken bonds are located in the middle part of the sample, except for the loading point. This pattern of broken bonds consistent with the fracture surface observed in the experiment results.

## Model Pile Installation in Maastricht Calcarenite

**Fig. 17:**
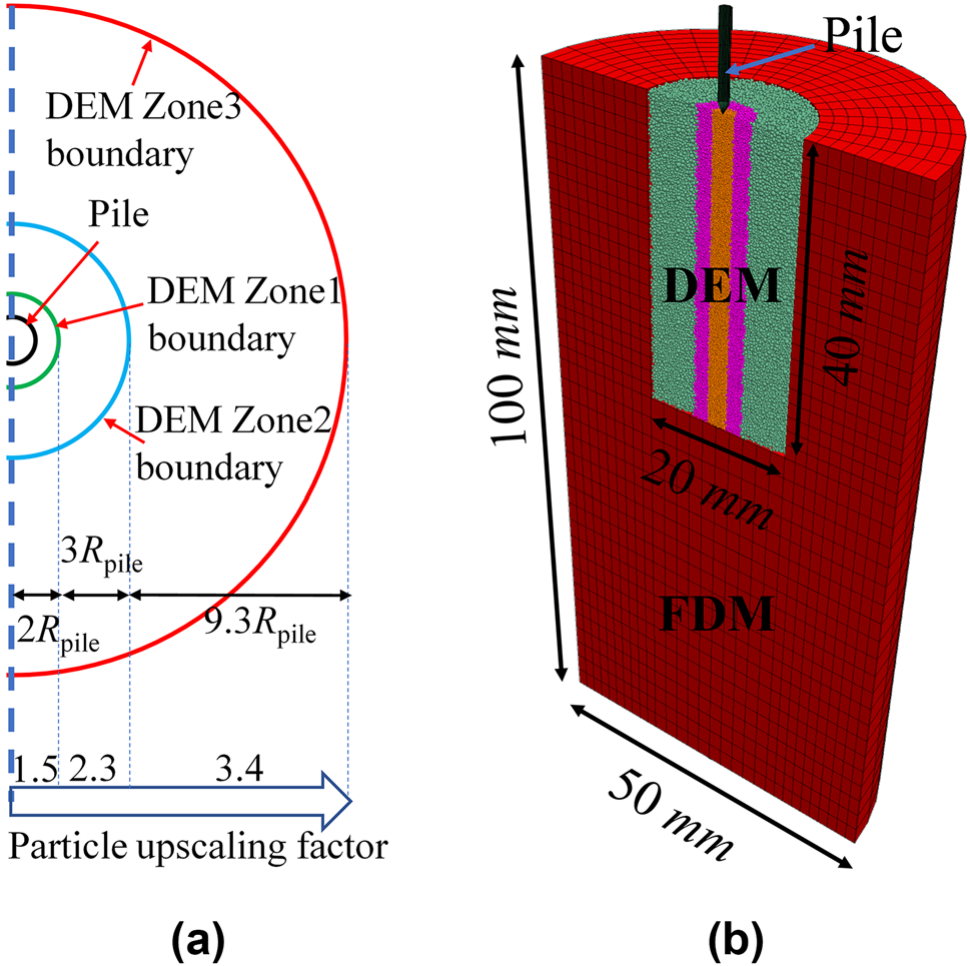
**a** Particle refinement upscaling factors in DEM region and **b** coupled model configuration

The efficiency of the proposed contact model is demonstrated by simulating the 1 g small-scale model pile installation tests in Maastricht calcarenite conducted by Ngan-Tillard et al. (2011). To improve computational efficiency whilst maintaining a domain size large enough to avoid boundary effects, the particle refinement method (McDowell and Falagush 2012) is adopted to have smaller particles near the pile shaft, larger ones as move away from it while a continuum FDM is used in the far field. The corresponding upscaling ratio, $S$, ranges from 1.5 to 3.4 in the DEM region. This is feasible as the model proposed is scale independent. Details on the DEM-FDM coupling method can be found in Zheng et al. (2022). In view of subsequent lateral loading, considering plane-symmetry across the pile, only half the domain is reproduced. Model dimensions are presented in Table 4. Contact parameters for the DEM region are the ones calibrated for the same calcarenite (Table 1). For the continuum part a linear Elastic model (Young modulus $E$ = 1.1 GPa and Poisson ration $\nu$ = 0.14) is used. The DEM region comprises about 195,000 particles, with 10,000 continuum elements in the continuum region, as shown in Fig. 17a. The contacts between the pile and particles are unbonded linear contacts, and the friction coefficient between the pile and calcarenite is 0.2 (Ziogos et al. 2023).

**Table 4:** Model information of the numerical coupled model

	Experiment	Full Model	DEM region
Chamber diameter (mm)	50	50	20
Chamber height (mm)	100	100	40
Model pile (mm)	1.4	1.4	/

$$n = 0.52$$, $$\rho = 2556\,{\mathrm{kg/m}}^{3}$$, $$K_{0} = 0.163$$, $$D_{{\mathrm{p}}} /d_{50} = 9$$

**Fig. 18:**
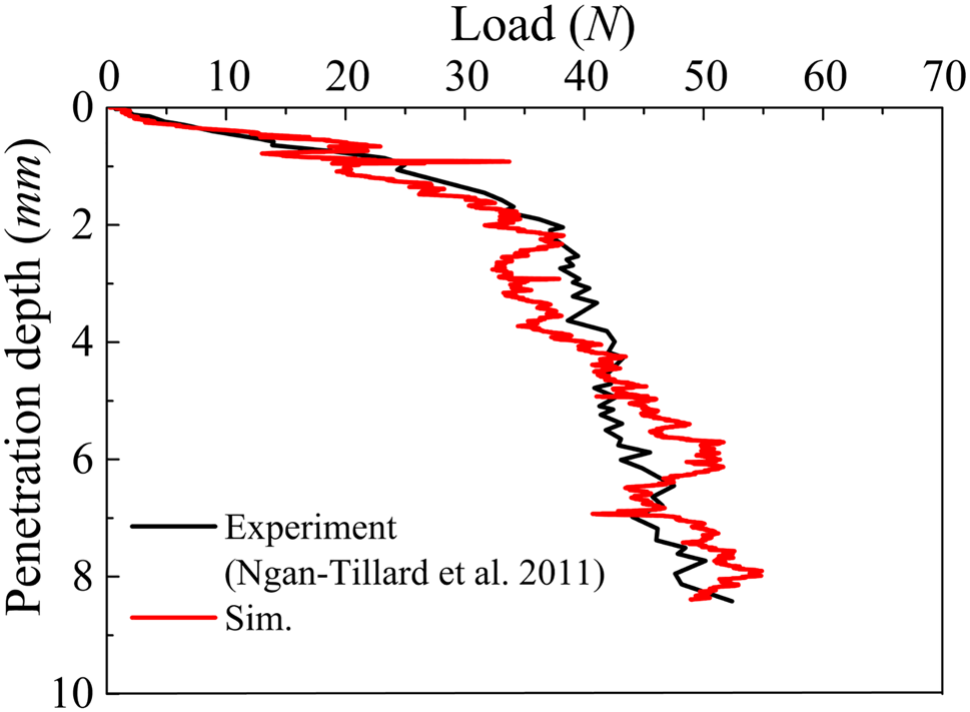
Comparison of experimental and numerical load–displacement curve during model pile installation in Maastricht calcarenite

The cone-shaped pile is penetrated to a final depth of 8.4 mm (pile diameter ${D}_{{\mathrm{p}}}$ = 1.4 mm) by jacking, the average installation velocity is 1.35 m/s to ensure local shearing strain rates are quasistatic (Ciantia et al. 2019a; Janda and Ooi 2016). As shown in Fig. 18 the model captures very well the experimental data in terms of load displacement. The simulation time to penetrate the model pile to a depth of 6 ${D}_{{\mathrm{p}}}$ is 4 days when using a standard desktop workstation (CPU @Intel i9-10980xe). Considering that similar continuum simulations using 2D-PFEM using axisymmetric conditions employ roughly 2–3 days for 10 ${D}_{{\mathrm{p}}}$ of penetration (Oliynyk et al. 2024), this result is promising.

**Fig. 19:**
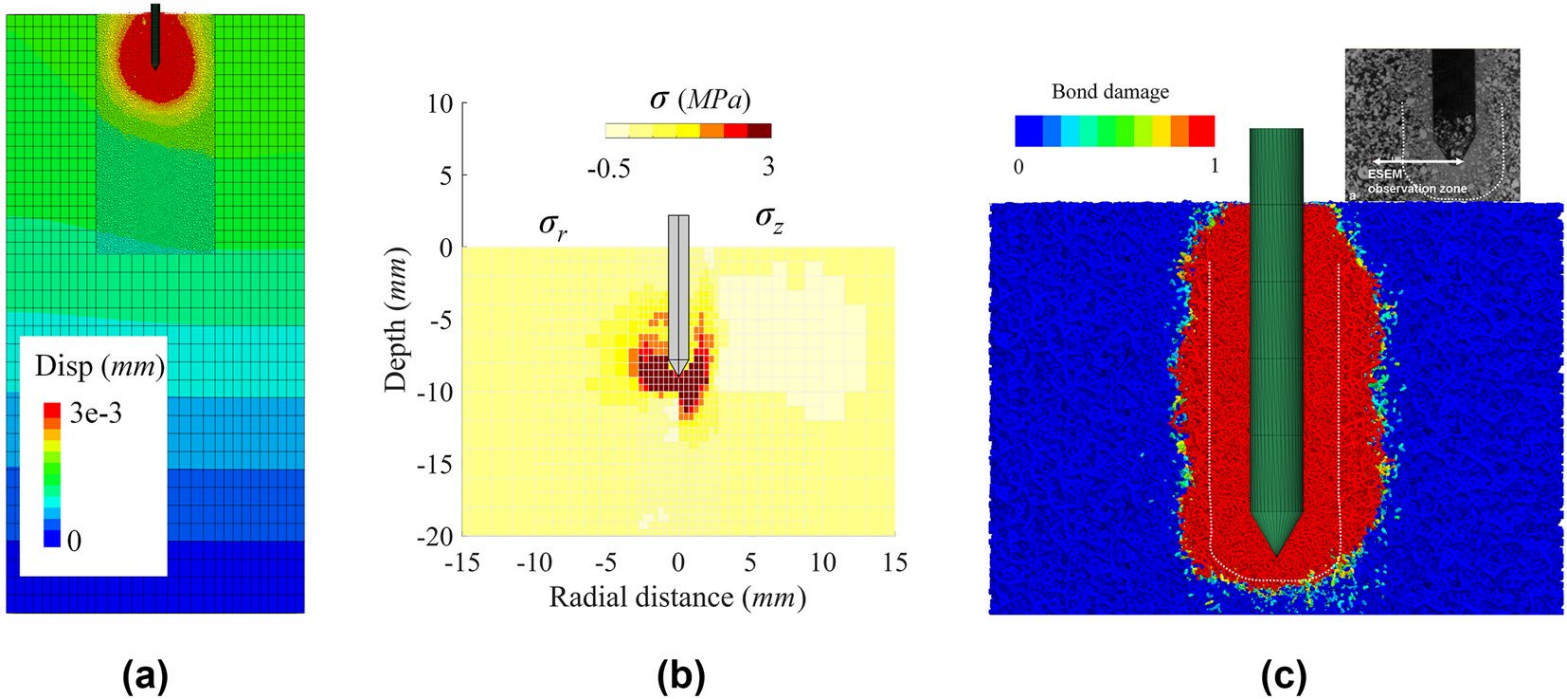
**a** Displacement field of the coupled model and **b** local stress distribution and **c** bond damage distribution after installation

Figure 19c illustrates the distribution of bond damage in the DEM region after installation. The blue force chains in Fig. 19c represent contact forces and are scaled by magnitude. The experimentally observed damage zone (green line) using X-Ray CT reported by Ngan-Tillard et al. (2011) is also reported to show that the predicted damage zone is very similar to what observed in the experiments. The simulations reveal that bond breakage develops in correspondence and below the pile tip while no damage occurs above the pile shoulder. Figure 19a, b depicts the displacement and local stress distributions at the end of pile installation obtained following the averaging procedures detailed in Ciantia et al. (2019a). The remarkable continuity of displacement and strain observed demonstrates the validity of the coupled modelling. Differently to what observed in granular materials, zones of tensile stress are seen to appear around the pile in cemented soft rocks. Similar stress profiles have also been observed in large deformation continuum models of cone ended pile installation (Oliynyk et al. 2021). Despite rock damage is quite localised, these stress variations involve larger regions and therefore boundary effects must be taken into consideration.

**Figure. 20:**
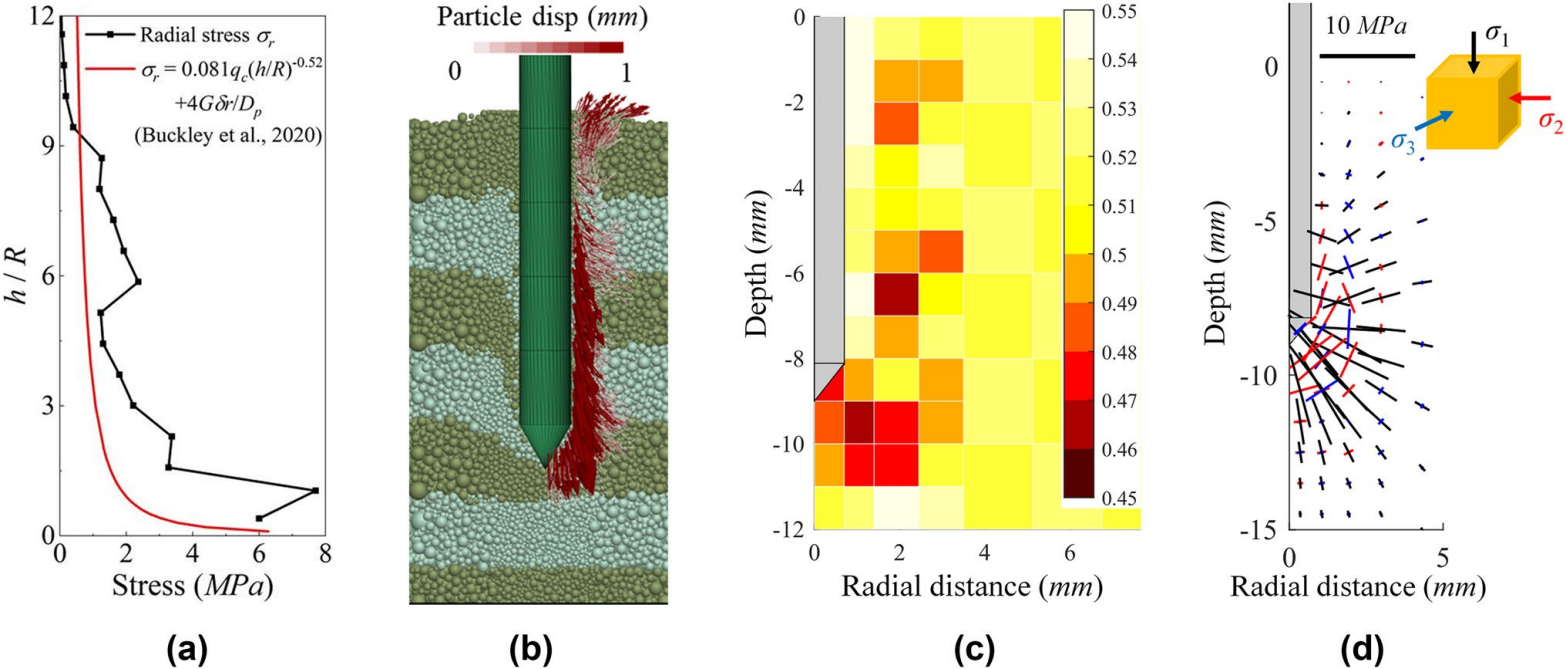
**a** Radial stress distribution along pile shaft; **b** particle movement; **c** porosity contour and **d** principal stress distribution

Figure 20a shows the radial stress decay on the pile shaft. Interestingly DEM trend is well captured by the recently proposed design method ICP-18 (Buckley et al. 2020) that was developed using large diameter pile filed tests in Chalk. In the field test by Buckley et al. (2020), and more recently in the model pile experiments by Riccio et al (2024), a shear band of crushed rock was observed forming around the pile shaft. An obvious shear band of completely crushed rock can be observed in Fig. 20b thanks to the micro nature of the DEM. On one side the figure shows the way particles displace as the pile penetrates and on the other the incremental displacement vectors. In Fig. 20b it is also possible to observe that ground layers become thinner below the pile tip. This is due to the collapse of the soft-rock. This is confirmed by the porosity contours plot shown in Fig. 20c. A densified crushed rock forms below the pile while a slight looser state is distributed around the pile shaft, probably caused by dilatant shearing. Finally, Fig. 20d depicts intense rotation of the principal stress axes near the pile, with the length of the axes representing stress magnitude. The maximum rotation of the major principal stress occurs near the pile shoulder, with a rotational angle of approximately 90°. Above the pile shoulder, all major principal stresses rotate toward the pile shaft, indicating the development of shaft resistance. Beneath the pile tip, the principal stress distribution reveals that the contact fabric aligns toward the pile tip. Combining this with the porosity distribution, it can be reasonably inferred that the rock beneath the pile tip undergoes vertical compression, while the rock near the shaft experiences radial compression during installation.

## Conclusion

In this study, we proposed a novel DEM contact model tailored to highly porous rocks, focusing on efficiency, scalability, and accuracy in capturing yielding and post-yield behaviour under various confinement conditions. The model introduces a far-field interaction concept, enabling the simulation of highly porous rocks using spherical particles, and is specifically designed to address boundary value problems. To describe the complex macroscopic transition of soft rocks from intact bonded states to fully remoulded (granular) states, the model is developed within a macro-element framework (combined loading) and incorporates damage-plasticity principles. Its validity and effectiveness are demonstrated across three scales: individual bonds, laboratory-scale samples, and large-scale boundary value problems (BVPs). The key conclusions are as follows:

(i) The complex transition of soft rocks from intact to completely remoulded states is governed by two competing effects: (a) softening caused by interparticle bond damage, and (b) hardening or softening (depending on confinement) induced by the densification or dilation frictional behaviour of the granular state.
(ii) Particle scaling is essential in large DEM models to reduce computational costs. The scalability feature of the proposed model allows calibration at one scale and application across various scales in the domain, akin to using a coarser mesh in continuum formulations. This ensures accurate responses without introducing numerical artifacts.
(iii) The model accommodates size effects, which are characteristic of rocks. It demonstrates how particle scaling and size effects can coexist and be incorporated into simulations when required.
(iv) The model's ability to address large-scale BVPs is demonstrated through a class A prediction of pile installation in Maastricht Calcarenite. Without requiring parameter tuning, the model accurately reproduces the force–displacement curve during pile installation. As the pile penetrates, the stress along the pile shaft decreases sharply upward from the pile tip, aligning closely with predictions from recently developed design methods. Leveraging its micromechanical foundation, the DEM model also provides insights into the underlying micro-mechanisms driving the observed macroscopic behaviour.

## Figures and Tables

**Fig. 21:**
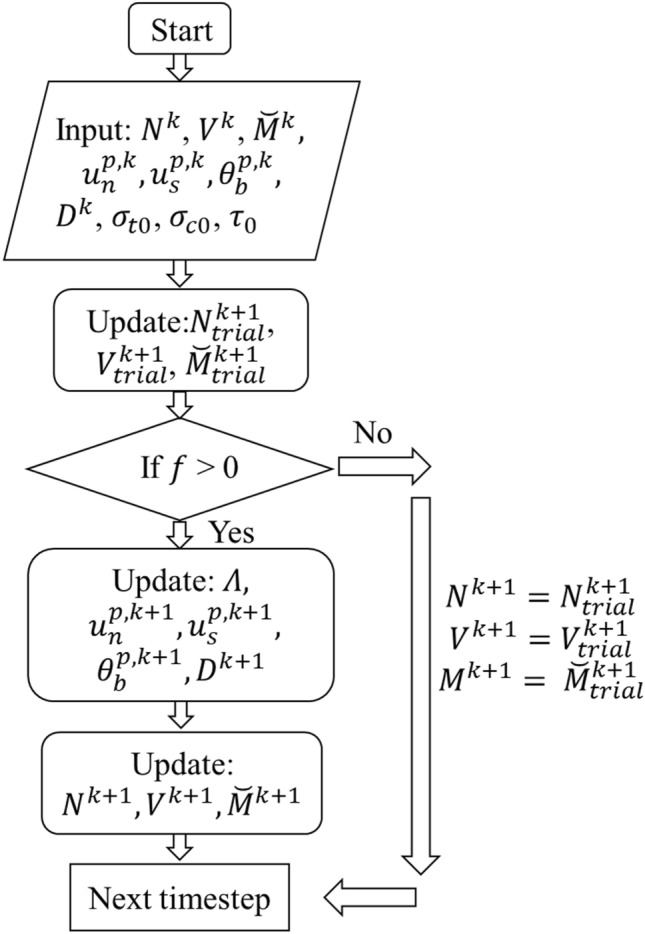
Schematic diagram of the update algorithm for the generalised load

**Fig. 22:**
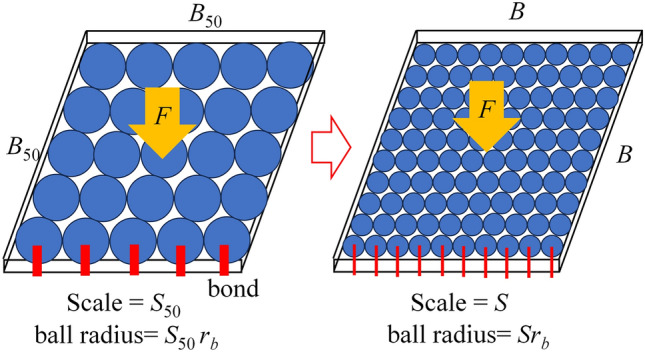
Particle upscaling independent theory

## Data Availability

Some or all data, models, or code that support the findings of this study are available from the corresponding author upon reasonable request.

## Appendix 1: Model Implementation Algorithm

The updated algorithm flow chart for the generalised load is presented in Fig. 21, the details are as follows.

*Step 1*: According to the calculation result of step $k$, and assuming that the deformation of step $k+1$ is elastic, the generalised trial load on the bond of step $k+1$ is calculated according to Eq. (9).

9 $$\left[\begin{array}{l}{N}_{{\mathrm{trial}}}^{k+1}\\ {V}_{{\mathrm{trial}}}^{k+1}\\ {\overset{\lower0.5em\hbox{$\smash{\scriptscriptstyle\smile}$}}{M}}_{{\mathrm{trial}}}^{k+1}\end{array}\right]=\left[\begin{array}{l}{N}^{k}+\left(1-D\right){k}_{{\mathrm{n}}}^{0}\delta {u}_{{\mathrm{n}}}^{k+1}A\\ {V}^{k}+\left(1-D\right){k}_{{\mathrm{s}}}^{0}\delta {u}_{{\mathrm{s}}}^{k+1}A\\ {\overset{\lower0.5em\hbox{$\smash{\scriptscriptstyle\smile}$}}{M}}^{k}+\left(1-D\right){k}_{{\mathrm{b}}}^{0}\delta {\theta }_{{\mathrm{b}}}^{k+1}/\overline{R}\end{array}\right]$$

*Step 2*: According to the trial generalised load calculated in the previous step, it is substituted into Eq. (5) to verify whether the yield function value is greater than 0.

*Step 3*: If the yield function value is greater than 0, it indicates that plastic deformation has occurred in step $k+1$. Based on the plasticity theory and the three-variable Taylor expansion, the plasticity multiplier is calculated as

10 $$\varLambda =\frac{-f}{\frac{\partial f}{\partial N}\frac{\partial N}{{\partial u}_{{\mathrm{n}}}^{p}}\frac{\partial g}{\partial N}+\frac{\partial f}{\partial V}\frac{\partial V}{{\partial u}_{{\mathrm{s}}}^{p}}\frac{\partial g}{\partial V}+\frac{\partial f}{\partial \overset{\lower0.5em\hbox{$\smash{\scriptscriptstyle\smile}$}}{M}}\frac{\partial \overset{\lower0.5em\hbox{$\smash{\scriptscriptstyle\smile}$}}{M}}{{\partial \theta }_{{\mathrm{b}}}^{p}}\frac{\partial g}{\partial \overset{\lower0.5em\hbox{$\smash{\scriptscriptstyle\smile}$}}{M}}+\left[\frac{\partial f}{\partial N}\frac{\partial N}{\partial D}+\frac{\partial f}{\partial V}\frac{\partial V}{\partial D}+\frac{\partial f}{\partial \overset{\lower0.5em\hbox{$\smash{\scriptscriptstyle\smile}$}}{M}}\frac{\partial \overset{\lower0.5em\hbox{$\smash{\scriptscriptstyle\smile}$}}{M}}{\partial D}\right]\left(\frac{\partial D}{{\partial u}_{{\mathrm{n}}}^{p}}\frac{\partial g}{\partial N}+\frac{\partial D}{{\partial u}_{{\mathrm{s}}}^{p}}\frac{\partial g}{\partial V}+\frac{\partial D}{{\partial \theta }_{{\mathrm{b}}}^{p}}\frac{\partial g}{\partial \overset{\lower0.5em\hbox{$\smash{\scriptscriptstyle\smile}$}}{M}}\right)}$$

This is an approximation method, and more details can be found in Nguyen et al. (2017).

Correspondingly, the plastic deformation increment and bond damage generated in step $k+1$ can be calculated according to Eqs. (7) and (3), respectively.

*Step 4*: Based on the plastic deformation and bond damage updated in the previous step, the true generalised load is obtained by

11 $$\left[\begin{array}{l}{N}^{k+1}\\ {V}^{k+1}\\ {\overset{\lower0.5em\hbox{$\smash{\scriptscriptstyle\smile}$}}{M}}^{k+1}\end{array}\right]=\left[\begin{array}{l}{N}_{{\mathrm{trial}}}^{k+1}\frac{1-{D}^{k+1}}{{1-D}^{k}}-\left(1-{D}^{k+1}\right){k}_{{\mathrm{n}}}^{0}\delta {u}_{{\mathrm{n}}}^{p,k+1}A\\ {V}_{{\mathrm{trial}}}^{k+1}\frac{1-{D}^{k+1}}{{1-D}^{k}}-\left(1-{D}^{k+1}\right){k}_{{\mathrm{n}}}^{0}\delta {u}_{{\mathrm{n}}}^{p,k+1}A\\ {\overset{\lower0.5em\hbox{$\smash{\scriptscriptstyle\smile}$}}{M}}_{{\mathrm{trial}}}^{k+1}\frac{1-{D}^{k+1}}{{1-D}^{k}}-\left(1-{D}^{k+1}\right){k}_{{\mathrm{n}}}^{0}\delta {u}_{{\mathrm{n}}}^{p,k+1}A/\overline{R}\end{array}\right]$$

## Appendix 2: Determination of the Closest Point on the Yield Surface

A combined Lagrange multiplier and Newton’s method with four variables is accepted here to find the specific closest point on the yield surface. Constructing a function $L$ as

12 $$\begin{aligned} L & = \left( {N_{x} - N_{{{\text{trial}}}}^{{k + 1}} } \right)^{2} + \left( {V_{x} - V_{{{\text{trial}}}}^{{k + 1}} } \right)^{2} + \left( {\overset{\lower0.5em\hbox{$\smash{\scriptscriptstyle\smile}$}}{M} _{x} - \overset{\lower0.5em\hbox{$\smash{\scriptscriptstyle\smile}$}}{M} _{{{\text{trial}}}}^{{k + 1}} } \right)^{2} \\ & \quad + \lambda \left( {\left( {\frac{{\overset{\lower0.5em\hbox{$\smash{\scriptscriptstyle\smile}$}}{M} _{x} }}{{\tilde{M}}}} \right)^{{1.001}} + \left( {\frac{{N_{x} }}{{\bar{N}}}} \right)^{2} + \left( {\frac{{\left( {\frac{{V_{x} }}{{\bar{V}}}} \right)^{4} }}{{1 - \left( {\frac{{N_{x} }}{{\bar{N}}}} \right)^{2} }}} \right) - 1} \right) = 0 \\ \end{aligned}$$

With $\lambda$ is the parameter in the Lagrange multiplier method.

The function $$L$$ is partially differentiated for the variables $${N}_{x}$$, $${V}_{x}$$, $${\overset{\lower0.5em\hbox{$\smash{\scriptscriptstyle\smile}$}}{M}}_{x}$$ and $$\lambda$$, respectively, four new functions are obtained.

13 $$\left\{\begin{array}{l}{L}_{1}=\frac{\partial L}{\partial {N}_{x}}=2\left({N}_{x}-{N}_{{\mathrm{trial}}}^{k+1}\right)+\lambda \left(\frac{2{N}_{x}}{{\overline{N} }^{2}}+\frac{2{N}_{x}{\overline{N} }^{2}{V}_{x}^{4}}{{\overline{V} }^{4}{\left({\overline{N} }^{2}-{N}_{x}^{2}\right)}^{2}}\right)=0\\ {L}_{2}=\frac{\partial L}{\partial {V}_{x}}=2({V}_{x}-{V}_{{\mathrm{trial}}}^{k+1})+\lambda \left(\frac{4{V}_{x}^{3}{\overline{N} }^{2}}{{\overline{V} }^{4}\left({\overline{N} }^{2}-{N}_{x}^{2}\right)}\right)=0\\ {L}_{3}=\frac{\partial L}{\partial {\overset{\lower0.5em\hbox{$\smash{\scriptscriptstyle\smile}$}}{M}}_{x}}=2({\overset{\lower0.5em\hbox{$\smash{\scriptscriptstyle\smile}$}}{M}}_{x}-{\overset{\lower0.5em\hbox{$\smash{\scriptscriptstyle\smile}$}}{M}}_{{\mathrm{trial}}}^{k+1})+\lambda \left(\frac{1.001{\overset{\lower0.5em\hbox{$\smash{\scriptscriptstyle\smile}$}}{M}}_{x}^{0.001}}{{\widetilde{M}}^{1.001}}\right)=0\\ {L}_{4}=\frac{\partial L}{\partial \lambda }={\left(\frac{{\overset{\lower0.5em\hbox{$\smash{\scriptscriptstyle\smile}$}}{M}}_{x}}{\widetilde{M}}\right)}^{1.001}+{\left(\frac{{N}_{x}}{\overline{N} }\right)}^{2}+\left(\frac{{\left(\frac{{V}_{x}}{\overline{V} }\right)}^{4}}{1-{\left(\frac{{N}_{x}}{\overline{N} }\right)}^{2}}\right)-1=0\end{array}\right.$$

Next, according to Newton's method, the variables$${N}_{x}$$,$${V}_{x}$$, $${\overset{\lower0.5em\hbox{$\smash{\scriptscriptstyle\smile}$}}{M}}_{x}$$ and $$\lambda$$ are partially differentiated for the above four functions to obtain a Jacobian matrix. Then inverting the Jacobian matrix and constructing the following equations according to the four-parameter Newton’s method. Finally, the coordinates of the closest point on the yield surface $$\left({N}_{x }, {V}_{x },{\overset{\lower0.5em\hbox{$\smash{\scriptscriptstyle\smile}$}}{M}}_{x}\right)$$ can be obtained by solving these equations.

14 $$\left[\begin{array}{l}{N}_{x}\\ {V}_{x}\\ {\overset{\lower0.5em\hbox{$\smash{\scriptscriptstyle\smile}$}}{M}}_{x}\\ \lambda \end{array}\right]=\left[\begin{array}{l}{N}_{x}\\ {V}_{x}\\ {\overset{\lower0.5em\hbox{$\smash{\scriptscriptstyle\smile}$}}{M}}_{x}\\ \lambda \end{array}\right]-{\left[\begin{array}{cccc}\frac{\partial {L}_{1}}{\partial {N}_{x}}& \frac{\partial {L}_{1}}{\partial {V}_{x}}& \frac{\partial {L}_{1}}{\partial {\overset{\lower0.5em\hbox{$\smash{\scriptscriptstyle\smile}$}}{M}}_{x}}& \frac{\partial {L}_{1}}{\partial \lambda }\\ \frac{\partial {L}_{2}}{\partial {N}_{x}}& \frac{\partial {L}_{2}}{\partial {V}_{x}}& \frac{\partial {L}_{2}}{\partial {\overset{\lower0.5em\hbox{$\smash{\scriptscriptstyle\smile}$}}{M}}_{x}}& \frac{\partial {L}_{2}}{\partial \lambda }\\ \frac{\partial {L}_{3}}{\partial {N}_{x}}& \frac{\partial {L}_{3}}{\partial {V}_{x}}& \frac{\partial {L}_{3}}{\partial {\overset{\lower0.5em\hbox{$\smash{\scriptscriptstyle\smile}$}}{M}}_{x}}& \frac{\partial {L}_{3}}{\partial \lambda }\\ \frac{\partial {L}_{4}}{\partial {N}_{x}}& \frac{\partial {L}_{4}}{\partial {V}_{x}}& \frac{\partial {L}_{4}}{\partial {\overset{\lower0.5em\hbox{$\smash{\scriptscriptstyle\smile}$}}{M}}_{x}}& \frac{\partial {L}_{4}}{\partial \lambda }\end{array}\right]}^{-1}\left[\begin{array}{l}{L}_{1}\\ {L}_{2}\\ {L}_{3}\\ {L}_{4}\end{array}\right]$$

## Appendix 3: Particle Upscaling Law Considering the Sample Size

Taking the schematic diagram of Fig. 22 under compression as an example, a relationship can be given as

15 $$\left\{ {\begin{array}{*{20}l} {F_{lim50} = UCS_{50} B_{50}^{2} = f_{n50,lim} N_{p50} = \sigma_{50} B_{50}^{2} } \\ {F_{lim} = UCSB^{2} = f_{n,lim} N_{{\mathrm{p}}} = \sigma B^{2} } \\ \end{array} } \right.$$

where note that the subscript 50 indicates that the properties of the sample with a characteristic (reference) width ($B_{50}$), i.e., the sample diameter is 50 mm. $F_{lim}$ is the limit normal force of the rock sample; $f_{n,lim}$ and $N_{{\mathrm{p}}}$ are the limited bonded normal force and the particle (or bond) number; $\sigma$ are the bond strengths in samples; $r_{{\mathrm{b}}}$ is the particle radius when the particle scale *S* = 1.

The schematic figure is an ideal case that does not consider the random particle packing and coordination number. To address it, a new parameter $\psi$ is introduced. Therefore, the above equation can be revised as

16 $$\left\{ {\begin{array}{*{20}l} {F_{lim50} = UCS_{50} B_{50}^{2} = \psi f_{n50,lim} N_{p50} = \sigma_{50} B_{50}^{2} } \\ {F_{lim} = UCSB^{2} = \psi f_{n,lim} N_{{\mathrm{p}}} = \sigma B^{2} } \\ \end{array} } \right.$$

Brown and Hoek (1980) proposed a relationship between the specimen size (diameter) and UCS, its form is presented as

17 $$UCS = UCS_{50} \left( \frac{50}{B} \right)^{0.18}$$

Combing the above two equations, then the bond strengths of samples with different $B$ can be expressed by

18 $$\sigma = \psi \sigma_{50} \left( \frac{50}{B} \right)^{0.18}$$

For the left softening and far-field interactions, their upscaling law incorporating the sample size effect is simply given based on Eq. (19):

19 $$\left\{ {\begin{array}{*{20}l} {u^{c} = \frac{S}{{S_{50} }}*u_{50}^{c} } \\ {\theta^{c} = \theta_{50}^{c} } \\ {g_{{\mathrm{a}}} = \frac{S}{{S_{50} }}*g_{a50} } \\ \end{array} } \right.$$

Six cubic samples with different sizes are generated to validate the upscaling law incorporating the sample size effect. The details and adopted model parameters of these six samples are summarized in Table 5. Left model parameters can be found in Table 2. The numerical sample with a diameter of 50 mm is the characteristic sample. For other samples, the strength, softening and the far-field interaction parameters are calculated based on the Eqs. (18) and (19), where $\psi$ is 1.1 calibrated based on the sample with a diameter of 10 mm.

**Table 5:** Model parameters for sample size effect related upscaling validation

Size (mm)	10 × 10 × 10	30 × 30 × 30	50 × 50 × 50	80 × 80 × 80	120 × 120 × 120	160 × 160 × 160
*S*	4	4, 12	20	12, 20, 32	48	48, 64
$N_{{\mathrm{p}}}$	13,465	13,465, 363,672	13,465	13,465, 55,166, 255,417	13,465	13,465, 31,923
$d_{00}$ (mm)	0.066 × *S*	0.066 × *S*	0.066 × *S*	0.066 × *S*	0.066 × *S*	0.066 × *S*
$\sigma_{{\mathrm{t}}}$ (MPa)	13.23	10.89	9	9.09	8.46	8.01
$\sigma_{{\mathrm{c}}}$ (MPa)	132.3	108.9	90	90.9	84.6	80.1
$C$ (MPa)	13.23	10.89	9	9.09	8.46	8.01
$u^{c}$ (m)	(1e−4) × *S*	(1e−4) × *S*	(1e−4) × *S*	(1e−4) × *S*	(1e−4) × *S*	(1e−4) × *S*
$\theta^{c}$ (rad)	1e−2	1e−2	1e−2	1e−2	1e−2	1e−2
$g_{{\mathrm{a}}}$ (mm)	0.1 $d_{00}$	0.1 $d_{00}$	0.1 $d_{00}$	0.1 $d_{00}$	0.1 $d_{00}$	0.1 $d_{00}$

## List of Symbols

$u_{{\mathrm{n}}} ,u_{{\mathrm{s}}}$: Normal and shear displacements of the bond
$\theta_{{\mathrm{b}}}$: Relative rotation of the bond
$u_{{\mathrm{n}}}^{e} ,u_{{\mathrm{s}}}^{e}$: Normal and shear elastic displacements of the bond
$\theta_{{\mathrm{b}}}^{e}$: Relative elastic rotation of the bond
$u_{{\mathrm{n}}}^{p} ,u_{{\mathrm{s}}}^{p}$: Normal and shear plastic displacements of the bond
$\theta_{{\mathrm{b}}}^{p}$: Relative plastic rotation of the bond
$$N,V,\overset{\lower0.5em\hbox{$\smash{\scriptscriptstyle\smile}$}}{M}$$: Generalised loads (normal and shear forces and normalised bending moment) on the bond
$$N_{{\mathrm{trial}}} ,V_{{\mathrm{trial}}} ,\overset{\lower0.5em\hbox{$\smash{\scriptscriptstyle\smile}$}}{M}_{{\mathrm{trial}}}$$: Trial generalised loads (normal and shear forces and normalised bending moment) on the bond
$$N_{x} ,V_{x} ,\overset{\lower0.5em\hbox{$\smash{\scriptscriptstyle\smile}$}}{M}_{x}$$: Coordinates of the closest point on the yield surface
$k_{{\mathrm{n}}}^{0} ,k_{{\mathrm{s}}}^{0} ,k_{{\mathrm{b}}}^{0}$: Initial normal, shear and bending stiffness of the bond
$k_{{\mathrm{n}}} ,k_{{\mathrm{s}}} ,k_{{\mathrm{b}}}$: Current normal, shear and bending stiffness of the bond
$A,\overline{R},l$: Area, radius and length of the bond
$I$: Inertia moment of the bond
$D$: Damage variable of the bond
$\overline{E}_{{\mathrm{mod}}} ,E_{{\mathrm{mod}}}$: Effective modulus of the bond and particle, respectively
$\overline{\kappa }^{*} ,\kappa^{*}$: Normal-to-shear stiffness of the bond and particle, respectively
$\overline{N},\overline{V},\overline{M}$: Parameters controlling the yield surface size of the bond
$\tilde{M}$: $\bar{M}$ normalised by bond radius.
$\sigma_{0}$: Normal strength of the bond
$\sigma_{{\mathrm{c}}} ,\sigma_{{\mathrm{t}}}$: Compressive and tensile strength of the bond
$C$: Cohesion of the bond
$f,g$: Yield function and plastic potential of the bond
$\delta u_{{\mathrm{n}}}^{p} ,\delta u_{{\mathrm{s}}}^{p}$: Incremental plastic displacements of the bond along normal and shear direction, respectively
$\delta \theta_{{\mathrm{b}}}^{p}$: Relative plastic rotation increment of the bond
$u^{c} ,\theta^{c}$: Parameters controlling the softening rate of the bond along translational and rotational direction
$g_{{\mathrm{b}}}$: Bond gap (bond activation gap)
$S$: Particle upscaling ratio
$F_{\lim } ,f_{n,\lim }$: Limit capacity of the sample and bond, respectively
$UCS$: Sample unconfined compressive strength value
$N_{{\mathrm{p}}} ,B$: Bond number and sample width (diameter), respectively
$\sigma$: Bond strength including $\sigma_{{\mathrm{c}}} ,\sigma_{{\mathrm{t}}}$ and $C$
$\psi$: Factor related to sample coordination number
$$\varLambda$$: Plastic multiplier
$\mu$: Frictional coefficient between particles
$g_{{\mathrm{a}}}$: Activation gap of the far-field interaction
$E,\nu$: Young’s modulus and Poisson ratio
$n,\rho$: The porosity and density
$K_{0}$: Lateral stress coefficient
$$D_{{\mathrm{p}}}$$: Pile diameter
$d_{00} ,d_{50}$: Minimum and mean diameter of particles
$p_{{\mathrm{a}}}$: Atmospheric pressure (101 kPa)
$p,q$: Mean and deviatoric stress
$\lambda$: Parameter in the Lagrange multiplier method
$$L$$: Lagrangian Function
$$L_{1} ,L_{2} ,L_{3} ,L_{4}$$: Four functions obtained by taking partial derivatives of the Lagrangian function *L* with respect to $$N_{x} ,V_{x} ,\overset{\lower0.5em\hbox{$\smash{\scriptscriptstyle\smile}$}}{M}_{x}$$ and $$\lambda$$, respectively
